# Supplementary figures and images for: Development of a prognostic model based on the ceRNA network in Triple-Negative Breast cancer
Source: PeerJ. 2025 Feb 27;13:e19063. doi: 10.7717/peerj.19063 (PMC11874946; doi:10.7717/peerj.19063)

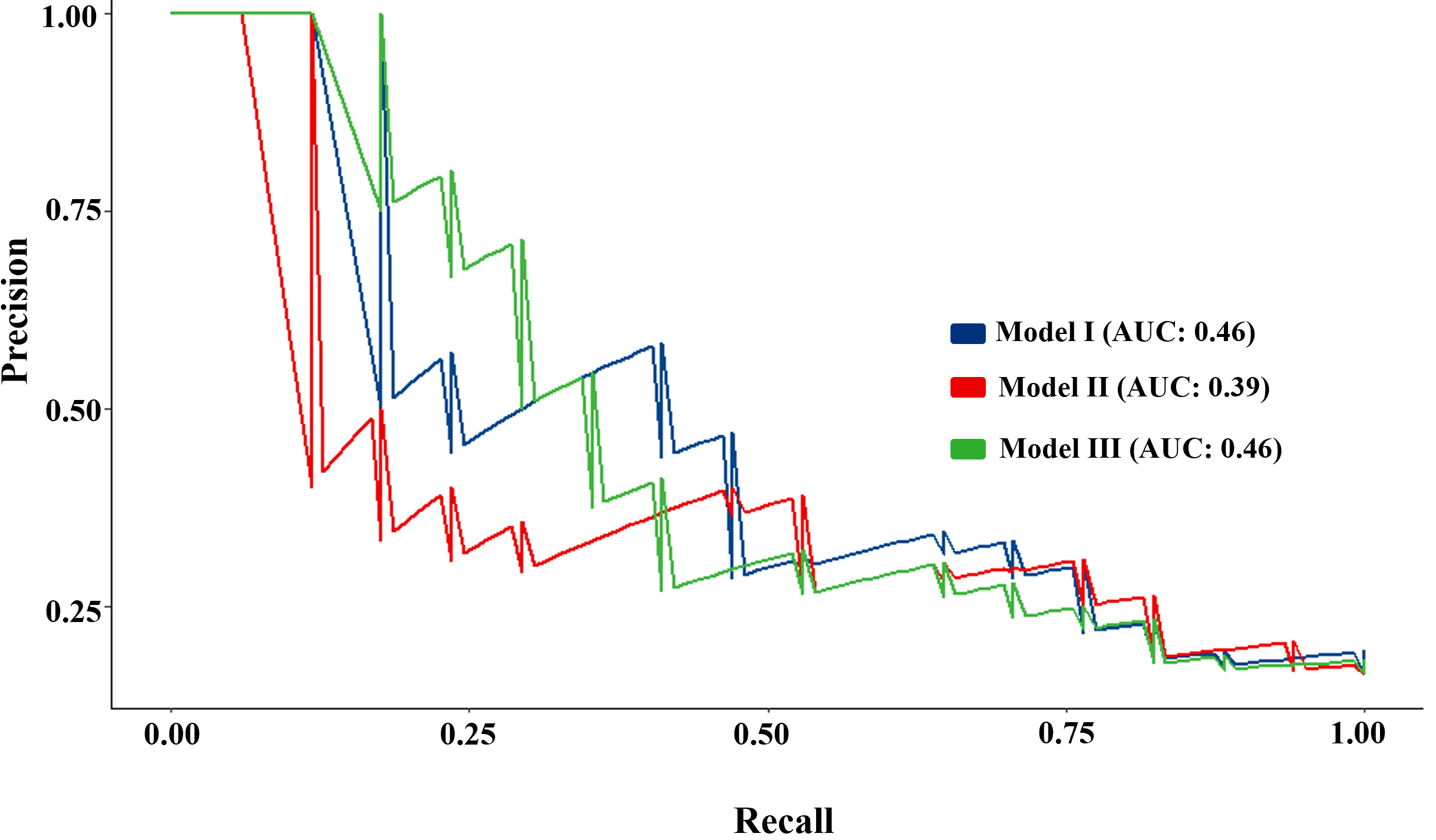

Supplement: Figure S1 [file peerj-13-19063-s006.tif]
