# Supplementary material for: Development of a prognostic model based on the ceRNA network in Triple-Negative Breast cancer
Source: PeerJ. 2025 Feb 27;13:e19063. doi: 10.7717/peerj.19063 (PMC11874946; doi:10.7717/peerj.19063)
Supplement: Table S1 [file peerj-13-19063-s007.docx]

**Table S1 The Relationships between lncRNA and and miRNA**

| **lncRNAs** | **miRNAs** |
| --- | --- |
| GRIK1-AS1 | hsa-miR-132 |
| GRIK1-AS1 | hsa-miR-212 |
| GRIK1-AS1 | hsa-miR-212-3p |
| GRIK1-AS1 | hsa-miR-138 |
| GRIK1-AS1 | hsa-miR-138ab |
| GRIK1-AS1 | hsa-miR-145 |
| GRIK1-AS1 | hsa-miR-199ab-5p |
| GRIK1-AS1 | hsa-miR-204 |
| GRIK1-AS1 | hsa-miR-204b |
| GRIK1-AS1 | hsa-miR-211 |
| GRIK1-AS1 | hsa-miR-205 |
| GRIK1-AS1 | hsa-miR-205ab |
| GRIK1-AS1 | hsa-miR-214 |
| GRIK1-AS1 | hsa-miR-761 |
| GRIK1-AS1 | hsa-miR-3619-5p |
| GRIK1-AS1 | hsa-miR-221 |
| GRIK1-AS1 | hsa-miR-222 |
| GRIK1-AS1 | hsa-miR-222ab |
| GRIK1-AS1 | hsa-miR-1928 |
| GRIK1-AS1 | hsa-miR-124 |
| GRIK1-AS1 | hsa-miR-124ab |
| GRIK1-AS1 | hsa-miR-506 |
| GRIK1-AS1 | hsa-miR-338 |
| GRIK1-AS1 | hsa-miR-338-3p |
| GRIK1-AS1 | hsa-miR-375 |
| GRIK1-AS1 | hsa-miR-383 |
| GRIK1-AS1 | hsa-miR-490-3p |
| WDFY3-AS2 | hsa-miR-7 |
| WDFY3-AS2 | hsa-miR-7ab |
| WDFY3-AS2 | hsa-miR-9 |
| WDFY3-AS2 | hsa-miR-9ab |
| WDFY3-AS2 | hsa-miR-96 |
| WDFY3-AS2 | hsa-miR-507 |
| WDFY3-AS2 | hsa-miR-1271 |
| WDFY3-AS2 | hsa-miR-135ab |
| WDFY3-AS2 | hsa-miR-135a-5p |
| WDFY3-AS2 | hsa-miR-138 |
| WDFY3-AS2 | hsa-miR-138ab |
| WDFY3-AS2 | hsa-miR-139-5p |
| WDFY3-AS2 | hsa-miR-141 |
| WDFY3-AS2 | hsa-miR-200a |
| WDFY3-AS2 | hsa-miR-142-3p |
| WDFY3-AS2 | hsa-miR-144 |
| WDFY3-AS2 | hsa-miR-145 |
| WDFY3-AS2 | hsa-miR-153 |
| WDFY3-AS2 | hsa-miR-155 |
| WDFY3-AS2 | hsa-miR-181abcd |
| WDFY3-AS2 | hsa-miR-4262 |
| WDFY3-AS2 | hsa-miR-182 |
| WDFY3-AS2 | hsa-miR-183 |
| WDFY3-AS2 | hsa-miR-let-7 |
| WDFY3-AS2 | hsa-miR-98 |
| WDFY3-AS2 | hsa-miR-4458 |
| WDFY3-AS2 | hsa-miR-4500 |
| WDFY3-AS2 | hsa-miR-18ab |
| WDFY3-AS2 | hsa-miR-4735-3p |
| WDFY3-AS2 | hsa-miR-190 |
| WDFY3-AS2 | hsa-miR-190ab |
| WDFY3-AS2 | hsa-miR-192 |
| WDFY3-AS2 | hsa-miR-215 |
| WDFY3-AS2 | hsa-miR-199ab-5p |
| WDFY3-AS2 | hsa-miR-19ab |
| WDFY3-AS2 | hsa-miR-1ab |
| WDFY3-AS2 | hsa-miR-206 |
| WDFY3-AS2 | hsa-miR-613 |
| WDFY3-AS2 | hsa-miR-200bc |
| WDFY3-AS2 | hsa-miR-429 |
| WDFY3-AS2 | hsa-miR-548a |
| WDFY3-AS2 | hsa-miR-21 |
| WDFY3-AS2 | hsa-miR-590-5p |
| WDFY3-AS2 | hsa-miR-217 |
| WDFY3-AS2 | hsa-miR-218 |
| WDFY3-AS2 | hsa-miR-218a |
| WDFY3-AS2 | hsa-miR-221 |
| WDFY3-AS2 | hsa-miR-222 |
| WDFY3-AS2 | hsa-miR-222ab |
| WDFY3-AS2 | hsa-miR-1928 |
| WDFY3-AS2 | hsa-miR-223 |
| WDFY3-AS2 | hsa-miR-23abc |
| WDFY3-AS2 | hsa-miR-23b-3p |
| WDFY3-AS2 | hsa-miR-24 |
| WDFY3-AS2 | hsa-miR-24ab |
| WDFY3-AS2 | hsa-miR-24-3p |
| WDFY3-AS2 | hsa-miR-26ab |
| WDFY3-AS2 | hsa-miR-1297 |
| WDFY3-AS2 | hsa-miR-4465 |
| WDFY3-AS2 | hsa-miR-27abc |
| WDFY3-AS2 | hsa-miR-27a-3p |
| WDFY3-AS2 | hsa-miR-101 |
| WDFY3-AS2 | hsa-miR-101ab |
| WDFY3-AS2 | hsa-miR-29abcd |
| WDFY3-AS2 | hsa-miR-31 |
| WDFY3-AS2 | hsa-miR-103a |
| WDFY3-AS2 | hsa-miR-107 |
| WDFY3-AS2 | hsa-miR-107ab |
| WDFY3-AS2 | hsa-miR-338 |
| WDFY3-AS2 | hsa-miR-338-3p |
| WDFY3-AS2 | hsa-miR-33a-3p |
| WDFY3-AS2 | hsa-miR-365 |
| WDFY3-AS2 | hsa-miR-365-3p |
| WDFY3-AS2 | hsa-miR-33ab |
| WDFY3-AS2 | hsa-miR-33-5p |
| WDFY3-AS2 | hsa-miR-34ac |
| WDFY3-AS2 | hsa-miR-34bc-5p |
| WDFY3-AS2 | hsa-miR-449abc |
| WDFY3-AS2 | hsa-miR-449c-5p |
| WDFY3-AS2 | hsa-miR-375 |
| WDFY3-AS2 | hsa-miR-425 |
| WDFY3-AS2 | hsa-miR-425-5p |
| WDFY3-AS2 | hsa-miR-489 |
| WDFY3-AS2 | hsa-miR-455-5p |
| WDFY3-AS2 | hsa-miR-128 |
| WDFY3-AS2 | hsa-miR-128ab |
| WDFY3-AS2 | hsa-miR-129-5p |
| WDFY3-AS2 | hsa-miR-129ab-5p |
| WDFY3-AS2 | hsa-miR-490-3p |
| MIR22HG | hsa-miR-133abc |
| MIR22HG | hsa-miR-199ab-5p |
| MIR22HG | hsa-miR-1ab |
| MIR22HG | hsa-miR-206 |
| MIR22HG | hsa-miR-613 |
| MIR22HG | hsa-miR-203 |
| MIR22HG | hsa-miR-24 |
| MIR22HG | hsa-miR-24ab |
| MIR22HG | hsa-miR-24-3p |
| MIR22HG | hsa-miR-25 |
| MIR22HG | hsa-miR-32 |
| MIR22HG | hsa-miR-92abc |
| MIR22HG | hsa-miR-363 |
| MIR22HG | hsa-miR-363-3p |
| MIR22HG | hsa-miR-367 |
| MIR22HG | hsa-miR-375 |
| MIR22HG | hsa-miR-383 |
| MIR22HG | hsa-miR-425 |
| MIR22HG | hsa-miR-425-5p |
| MIR22HG | hsa-miR-489 |
| SNHG12 | hsa-miR-503 |
| SNHG12 | hsa-miR-133abc |
| SNHG12 | hsa-miR-9 |
| SNHG12 | hsa-miR-9ab |
| SNHG12 | hsa-miR-138 |
| SNHG12 | hsa-miR-138ab |
| SNHG12 | hsa-miR-140 |
| SNHG12 | hsa-miR-140-5p |
| SNHG12 | hsa-miR-876-3p |
| SNHG12 | hsa-miR-1244 |
| SNHG12 | hsa-miR-146ac |
| SNHG12 | hsa-miR-146b-5p |
| SNHG12 | hsa-miR-148ab-3p |
| SNHG12 | hsa-miR-152 |
| SNHG12 | hsa-miR-150 |
| SNHG12 | hsa-miR-5127 |
| SNHG12 | hsa-miR-15abc |
| SNHG12 | hsa-miR-16 |
| SNHG12 | hsa-miR-16abc |
| SNHG12 | hsa-miR-195 |
| SNHG12 | hsa-miR-322 |
| SNHG12 | hsa-miR-424 |
| SNHG12 | hsa-miR-497 |
| SNHG12 | hsa-miR-1907 |
| SNHG12 | hsa-miR-181abcd |
| SNHG12 | hsa-miR-4262 |
| SNHG12 | hsa-miR-let-7 |
| SNHG12 | hsa-miR-98 |
| SNHG12 | hsa-miR-4458 |
| SNHG12 | hsa-miR-4500 |
| SNHG12 | hsa-miR-187 |
| SNHG12 | hsa-miR-193 |
| SNHG12 | hsa-miR-193b |
| SNHG12 | hsa-miR-193a-3p |
| SNHG12 | hsa-miR-194 |
| SNHG12 | hsa-miR-199ab-5p |
| SNHG12 | hsa-miR-1ab |
| SNHG12 | hsa-miR-206 |
| SNHG12 | hsa-miR-613 |
| SNHG12 | hsa-miR-200bc |
| SNHG12 | hsa-miR-429 |
| SNHG12 | hsa-miR-548a |
| SNHG12 | hsa-miR-204 |
| SNHG12 | hsa-miR-204b |
| SNHG12 | hsa-miR-211 |
| SNHG12 | hsa-miR-208ab |
| SNHG12 | hsa-miR-208ab-3p |
| SNHG12 | hsa-miR-217 |
| SNHG12 | hsa-miR-218 |
| SNHG12 | hsa-miR-218a |
| SNHG12 | hsa-miR-219-5p |
| SNHG12 | hsa-miR-508 |
| SNHG12 | hsa-miR-508-3p |
| SNHG12 | hsa-miR-4782-3p |
| SNHG12 | hsa-miR-122 |
| SNHG12 | hsa-miR-122a |
| SNHG12 | hsa-miR-1352 |
| SNHG12 | hsa-miR-24 |
| SNHG12 | hsa-miR-24ab |
| SNHG12 | hsa-miR-24-3p |
| SNHG12 | hsa-miR-338 |
| SNHG12 | hsa-miR-338-3p |
| SNHG12 | hsa-miR-33ab |
| SNHG12 | hsa-miR-33-5p |
| SNHG12 | hsa-miR-425 |
| SNHG12 | hsa-miR-425-5p |
| SNHG12 | hsa-miR-489 |
| SNHG12 | hsa-miR-10abc |
| SNHG12 | hsa-miR-10a-5p |
| SNHG12 | hsa-miR-451 |
| SNHG12 | hsa-miR-129-5p |
| SNHG12 | hsa-miR-129ab-5p |
| SNHG12 | hsa-miR-499-5p |
| MEG3 | hsa-miR-551a |
| MEG3 | hsa-miR-130ac |
| MEG3 | hsa-miR-301ab |
| MEG3 | hsa-miR-301b |
| MEG3 | hsa-miR-301b-3p |
| MEG3 | hsa-miR-454 |
| MEG3 | hsa-miR-721 |
| MEG3 | hsa-miR-4295 |
| MEG3 | hsa-miR-3666 |
| MEG3 | hsa-miR-132 |
| MEG3 | hsa-miR-212 |
| MEG3 | hsa-miR-212-3p |
| MEG3 | hsa-miR-7 |
| MEG3 | hsa-miR-7ab |
| MEG3 | hsa-miR-133abc |
| MEG3 | hsa-miR-9 |
| MEG3 | hsa-miR-9ab |
| MEG3 | hsa-miR-93 |
| MEG3 | hsa-miR-93a |
| MEG3 | hsa-miR-105 |
| MEG3 | hsa-miR-106a |
| MEG3 | hsa-miR-291a-3p |
| MEG3 | hsa-miR-294 |
| MEG3 | hsa-miR-295 |
| MEG3 | hsa-miR-302abcde |
| MEG3 | hsa-miR-372 |
| MEG3 | hsa-miR-373 |
| MEG3 | hsa-miR-428 |
| MEG3 | hsa-miR-519a |
| MEG3 | hsa-miR-520be |
| MEG3 | hsa-miR-520acd-3p |
| MEG3 | hsa-miR-1378 |
| MEG3 | hsa-miR-1420ac |
| MEG3 | hsa-miR-96 |
| MEG3 | hsa-miR-507 |
| MEG3 | hsa-miR-1271 |
| MEG3 | hsa-miR-135ab |
| MEG3 | hsa-miR-135a-5p |
| MEG3 | hsa-miR-138 |
| MEG3 | hsa-miR-138ab |
| MEG3 | hsa-miR-140 |
| MEG3 | hsa-miR-140-5p |
| MEG3 | hsa-miR-876-3p |
| MEG3 | hsa-miR-1244 |
| MEG3 | hsa-miR-141 |
| MEG3 | hsa-miR-200a |
| MEG3 | hsa-miR-142-3p |
| MEG3 | hsa-miR-143 |
| MEG3 | hsa-miR-1721 |
| MEG3 | hsa-miR-4770 |
| MEG3 | hsa-miR-144 |
| MEG3 | hsa-miR-145 |
| MEG3 | hsa-miR-150 |
| MEG3 | hsa-miR-5127 |
| MEG3 | hsa-miR-155 |
| MEG3 | hsa-miR-15abc |
| MEG3 | hsa-miR-16 |
| MEG3 | hsa-miR-16abc |
| MEG3 | hsa-miR-195 |
| MEG3 | hsa-miR-322 |
| MEG3 | hsa-miR-424 |
| MEG3 | hsa-miR-497 |
| MEG3 | hsa-miR-1907 |
| MEG3 | hsa-miR-17 |
| MEG3 | hsa-miR-17-5p |
| MEG3 | hsa-miR-20ab |
| MEG3 | hsa-miR-20b-5p |
| MEG3 | hsa-miR-106ab |
| MEG3 | hsa-miR-427 |
| MEG3 | hsa-miR-518a-3p |
| MEG3 | hsa-miR-519d |
| MEG3 | hsa-miR-181abcd |
| MEG3 | hsa-miR-4262 |
| MEG3 | hsa-miR-182 |
| MEG3 | hsa-miR-184 |
| MEG3 | hsa-miR-let-7 |
| MEG3 | hsa-miR-98 |
| MEG3 | hsa-miR-4458 |
| MEG3 | hsa-miR-4500 |
| MEG3 | hsa-miR-18ab |
| MEG3 | hsa-miR-4735-3p |
| MEG3 | hsa-miR-192 |
| MEG3 | hsa-miR-215 |
| MEG3 | hsa-miR-199ab-5p |
| MEG3 | hsa-miR-19ab |
| MEG3 | hsa-miR-1ab |
| MEG3 | hsa-miR-206 |
| MEG3 | hsa-miR-613 |
| MEG3 | hsa-miR-200bc |
| MEG3 | hsa-miR-429 |
| MEG3 | hsa-miR-548a |
| MEG3 | hsa-miR-203 |
| MEG3 | hsa-miR-204 |
| MEG3 | hsa-miR-204b |
| MEG3 | hsa-miR-211 |
| MEG3 | hsa-miR-205 |
| MEG3 | hsa-miR-205ab |
| MEG3 | hsa-miR-208ab |
| MEG3 | hsa-miR-208ab-3p |
| MEG3 | hsa-miR-21 |
| MEG3 | hsa-miR-590-5p |
| MEG3 | hsa-miR-214 |
| MEG3 | hsa-miR-761 |
| MEG3 | hsa-miR-3619-5p |
| MEG3 | hsa-miR-216a |
| MEG3 | hsa-miR-216b |
| MEG3 | hsa-miR-216b-5p |
| MEG3 | hsa-miR-217 |
| MEG3 | hsa-miR-218 |
| MEG3 | hsa-miR-218a |
| MEG3 | hsa-miR-219-5p |
| MEG3 | hsa-miR-508 |
| MEG3 | hsa-miR-508-3p |
| MEG3 | hsa-miR-4782-3p |
| MEG3 | hsa-miR-22 |
| MEG3 | hsa-miR-22-3p |
| MEG3 | hsa-miR-221 |
| MEG3 | hsa-miR-222 |
| MEG3 | hsa-miR-222ab |
| MEG3 | hsa-miR-1928 |
| MEG3 | hsa-miR-223 |
| MEG3 | hsa-miR-122 |
| MEG3 | hsa-miR-122a |
| MEG3 | hsa-miR-1352 |
| MEG3 | hsa-miR-23abc |
| MEG3 | hsa-miR-23b-3p |
| MEG3 | hsa-miR-24 |
| MEG3 | hsa-miR-24ab |
| MEG3 | hsa-miR-24-3p |
| MEG3 | hsa-miR-26ab |
| MEG3 | hsa-miR-1297 |
| MEG3 | hsa-miR-4465 |
| MEG3 | hsa-miR-27abc |
| MEG3 | hsa-miR-27a-3p |
| MEG3 | hsa-miR-31 |
| MEG3 | hsa-miR-103a |
| MEG3 | hsa-miR-107 |
| MEG3 | hsa-miR-107ab |
| MEG3 | hsa-miR-124 |
| MEG3 | hsa-miR-124ab |
| MEG3 | hsa-miR-506 |
| MEG3 | hsa-miR-338 |
| MEG3 | hsa-miR-338-3p |
| MEG3 | hsa-miR-33a-3p |
| MEG3 | hsa-miR-365 |
| MEG3 | hsa-miR-365-3p |
| MEG3 | hsa-miR-34ac |
| MEG3 | hsa-miR-34bc-5p |
| MEG3 | hsa-miR-449abc |
| MEG3 | hsa-miR-449c-5p |
| MEG3 | hsa-miR-383 |
| MEG3 | hsa-miR-125a-5p |
| MEG3 | hsa-miR-125b-5p |
| MEG3 | hsa-miR-351 |
| MEG3 | hsa-miR-670 |
| MEG3 | hsa-miR-4319 |
| MEG3 | hsa-miR-10abc |
| MEG3 | hsa-miR-10a-5p |
| MEG3 | hsa-miR-451 |
| MEG3 | hsa-miR-455-5p |
| MEG3 | hsa-miR-128 |
| MEG3 | hsa-miR-128ab |
| MEG3 | hsa-miR-129-5p |
| MEG3 | hsa-miR-129ab-5p |
| MEG3 | hsa-miR-490-3p |
| MEG3 | hsa-miR-499-5p |
| MCM3AP-AS1 | hsa-miR-503 |
| MCM3AP-AS1 | hsa-miR-130ac |
| MCM3AP-AS1 | hsa-miR-301ab |
| MCM3AP-AS1 | hsa-miR-301b |
| MCM3AP-AS1 | hsa-miR-301b-3p |
| MCM3AP-AS1 | hsa-miR-454 |
| MCM3AP-AS1 | hsa-miR-721 |
| MCM3AP-AS1 | hsa-miR-4295 |
| MCM3AP-AS1 | hsa-miR-3666 |
| MCM3AP-AS1 | hsa-miR-132 |
| MCM3AP-AS1 | hsa-miR-212 |
| MCM3AP-AS1 | hsa-miR-212-3p |
| MCM3AP-AS1 | hsa-miR-7 |
| MCM3AP-AS1 | hsa-miR-7ab |
| MCM3AP-AS1 | hsa-miR-96 |
| MCM3AP-AS1 | hsa-miR-507 |
| MCM3AP-AS1 | hsa-miR-1271 |
| MCM3AP-AS1 | hsa-miR-137 |
| MCM3AP-AS1 | hsa-miR-137ab |
| MCM3AP-AS1 | hsa-miR-138 |
| MCM3AP-AS1 | hsa-miR-138ab |
| MCM3AP-AS1 | hsa-miR-139-5p |
| MCM3AP-AS1 | hsa-miR-140 |
| MCM3AP-AS1 | hsa-miR-140-5p |
| MCM3AP-AS1 | hsa-miR-876-3p |
| MCM3AP-AS1 | hsa-miR-1244 |
| MCM3AP-AS1 | hsa-miR-141 |
| MCM3AP-AS1 | hsa-miR-200a |
| MCM3AP-AS1 | hsa-miR-142-3p |
| MCM3AP-AS1 | hsa-miR-143 |
| MCM3AP-AS1 | hsa-miR-1721 |
| MCM3AP-AS1 | hsa-miR-4770 |
| MCM3AP-AS1 | hsa-miR-144 |
| MCM3AP-AS1 | hsa-miR-146ac |
| MCM3AP-AS1 | hsa-miR-146b-5p |
| MCM3AP-AS1 | hsa-miR-148ab-3p |
| MCM3AP-AS1 | hsa-miR-152 |
| MCM3AP-AS1 | hsa-miR-150 |
| MCM3AP-AS1 | hsa-miR-5127 |
| MCM3AP-AS1 | hsa-miR-153 |
| MCM3AP-AS1 | hsa-miR-155 |
| MCM3AP-AS1 | hsa-miR-15abc |
| MCM3AP-AS1 | hsa-miR-16 |
| MCM3AP-AS1 | hsa-miR-16abc |
| MCM3AP-AS1 | hsa-miR-195 |
| MCM3AP-AS1 | hsa-miR-322 |
| MCM3AP-AS1 | hsa-miR-424 |
| MCM3AP-AS1 | hsa-miR-497 |
| MCM3AP-AS1 | hsa-miR-1907 |
| MCM3AP-AS1 | hsa-miR-182 |
| MCM3AP-AS1 | hsa-miR-let-7 |
| MCM3AP-AS1 | hsa-miR-98 |
| MCM3AP-AS1 | hsa-miR-4458 |
| MCM3AP-AS1 | hsa-miR-4500 |
| MCM3AP-AS1 | hsa-miR-187 |
| MCM3AP-AS1 | hsa-miR-18ab |
| MCM3AP-AS1 | hsa-miR-4735-3p |
| MCM3AP-AS1 | hsa-miR-190 |
| MCM3AP-AS1 | hsa-miR-190ab |
| MCM3AP-AS1 | hsa-miR-192 |
| MCM3AP-AS1 | hsa-miR-215 |
| MCM3AP-AS1 | hsa-miR-194 |
| MCM3AP-AS1 | hsa-miR-19ab |
| MCM3AP-AS1 | hsa-miR-1ab |
| MCM3AP-AS1 | hsa-miR-206 |
| MCM3AP-AS1 | hsa-miR-613 |
| MCM3AP-AS1 | hsa-miR-200bc |
| MCM3AP-AS1 | hsa-miR-429 |
| MCM3AP-AS1 | hsa-miR-548a |
| MCM3AP-AS1 | hsa-miR-203 |
| MCM3AP-AS1 | hsa-miR-204 |
| MCM3AP-AS1 | hsa-miR-204b |
| MCM3AP-AS1 | hsa-miR-211 |
| MCM3AP-AS1 | hsa-miR-205 |
| MCM3AP-AS1 | hsa-miR-205ab |
| MCM3AP-AS1 | hsa-miR-21 |
| MCM3AP-AS1 | hsa-miR-590-5p |
| MCM3AP-AS1 | hsa-miR-214 |
| MCM3AP-AS1 | hsa-miR-761 |
| MCM3AP-AS1 | hsa-miR-3619-5p |
| MCM3AP-AS1 | hsa-miR-217 |
| MCM3AP-AS1 | hsa-miR-219-5p |
| MCM3AP-AS1 | hsa-miR-508 |
| MCM3AP-AS1 | hsa-miR-508-3p |
| MCM3AP-AS1 | hsa-miR-4782-3p |
| MCM3AP-AS1 | hsa-miR-22 |
| MCM3AP-AS1 | hsa-miR-22-3p |
| MCM3AP-AS1 | hsa-miR-223 |
| MCM3AP-AS1 | hsa-miR-122 |
| MCM3AP-AS1 | hsa-miR-122a |
| MCM3AP-AS1 | hsa-miR-1352 |
| MCM3AP-AS1 | hsa-miR-23abc |
| MCM3AP-AS1 | hsa-miR-23b-3p |
| MCM3AP-AS1 | hsa-miR-24 |
| MCM3AP-AS1 | hsa-miR-24ab |
| MCM3AP-AS1 | hsa-miR-24-3p |
| MCM3AP-AS1 | hsa-miR-101 |
| MCM3AP-AS1 | hsa-miR-101ab |
| MCM3AP-AS1 | hsa-miR-30abcdef |
| MCM3AP-AS1 | hsa-miR-30abe-5p |
| MCM3AP-AS1 | hsa-miR-384-5p |
| MCM3AP-AS1 | hsa-miR-338 |
| MCM3AP-AS1 | hsa-miR-338-3p |
| MCM3AP-AS1 | hsa-miR-33a-3p |
| MCM3AP-AS1 | hsa-miR-365 |
| MCM3AP-AS1 | hsa-miR-365-3p |
| MCM3AP-AS1 | hsa-miR-383 |
| MCM3AP-AS1 | hsa-miR-425 |
| MCM3AP-AS1 | hsa-miR-425-5p |
| MCM3AP-AS1 | hsa-miR-489 |
| MCM3AP-AS1 | hsa-miR-125a-5p |
| MCM3AP-AS1 | hsa-miR-125b-5p |
| MCM3AP-AS1 | hsa-miR-351 |
| MCM3AP-AS1 | hsa-miR-670 |
| MCM3AP-AS1 | hsa-miR-4319 |
| MCM3AP-AS1 | hsa-miR-455-5p |
| MCM3AP-AS1 | hsa-miR-129-5p |
| MCM3AP-AS1 | hsa-miR-129ab-5p |
| MCM3AP-AS1 | hsa-miR-499-5p |
| THRB-IT1 | hsa-miR-133abc |
| THRB-IT1 | hsa-miR-139-5p |
| THRB-IT1 | hsa-miR-146ac |
| THRB-IT1 | hsa-miR-146b-5p |
| THRB-IT1 | hsa-miR-148ab-3p |
| THRB-IT1 | hsa-miR-152 |
| lncRNA | miRNA |
| THRB-IT1 | hsa-miR-153 |
| THRB-IT1 | hsa-miR-155 |
| THRB-IT1 | hsa-miR-181abcd |
| THRB-IT1 | hsa-miR-4262 |
| THRB-IT1 | hsa-miR-let-7 |
| THRB-IT1 | hsa-miR-98 |
| THRB-IT1 | hsa-miR-4458 |
| THRB-IT1 | hsa-miR-4500 |
| THRB-IT1 | hsa-miR-1ab |
| THRB-IT1 | hsa-miR-206 |
| THRB-IT1 | hsa-miR-613 |
| THRB-IT1 | hsa-miR-200bc |
| THRB-IT1 | hsa-miR-429 |
| THRB-IT1 | hsa-miR-548a |
| THRB-IT1 | hsa-miR-203 |
| THRB-IT1 | hsa-miR-204 |
| THRB-IT1 | hsa-miR-204b |
| THRB-IT1 | hsa-miR-211 |
| THRB-IT1 | hsa-miR-216a |
| THRB-IT1 | hsa-miR-216b |
| THRB-IT1 | hsa-miR-216b-5p |
| THRB-IT1 | hsa-miR-217 |
| THRB-IT1 | hsa-miR-23abc |
| THRB-IT1 | hsa-miR-23b-3p |
| THRB-IT1 | hsa-miR-103a |
| THRB-IT1 | hsa-miR-107 |
| THRB-IT1 | hsa-miR-107ab |
| THRB-IT1 | hsa-miR-10abc |
| THRB-IT1 | hsa-miR-10a-5p |
| THRB-IT1 | hsa-miR-455-5p |
| LINC00092 | hsa-miR-150 |
| LINC00092 | hsa-miR-5127 |
| LINC00092 | hsa-miR-15abc |
| LINC00092 | hsa-miR-16 |
| LINC00092 | hsa-miR-16abc |
| LINC00092 | hsa-miR-195 |
| LINC00092 | hsa-miR-322 |
| LINC00092 | hsa-miR-424 |
| LINC00092 | hsa-miR-497 |
| LINC00092 | hsa-miR-1907 |
| LINC00092 | hsa-miR-184 |
| LINC00092 | hsa-miR-1ab |
| LINC00092 | hsa-miR-206 |
| LINC00092 | hsa-miR-613 |
| LINC00092 | hsa-miR-33a-3p |
| LINC00092 | hsa-miR-365 |
| LINC00092 | hsa-miR-365-3p |
| LINC00113 | hsa-miR-135ab |
| LINC00113 | hsa-miR-135a-5p |
| LINC00113 | hsa-miR-145 |
| LINC00113 | hsa-miR-150 |
| LINC00113 | hsa-miR-5127 |
| LINC00113 | hsa-miR-18ab |
| LINC00113 | hsa-miR-4735-3p |
| LINC00113 | hsa-miR-203 |
| LINC00113 | hsa-miR-29abcd |
| LINC00113 | hsa-miR-338 |
| LINC00113 | hsa-miR-338-3p |
| MAST4-AS1 | hsa-miR-9 |
| MAST4-AS1 | hsa-miR-9ab |
| MAST4-AS1 | hsa-miR-205 |
| MAST4-AS1 | hsa-miR-205ab |
| MAST4-AS1 | hsa-miR-23abc |
| MAST4-AS1 | hsa-miR-23b-3p |
| MAST4-AS1 | hsa-miR-26ab |
| MAST4-AS1 | hsa-miR-1297 |
| MAST4-AS1 | hsa-miR-4465 |
| MAST4-AS1 | hsa-miR-30abcdef |
| MAST4-AS1 | hsa-miR-30abe-5p |
| MAST4-AS1 | hsa-miR-384-5p |
| EMX2OS | hsa-miR-503 |
| EMX2OS | hsa-miR-138 |
| EMX2OS | hsa-miR-138ab |
| EMX2OS | hsa-miR-143 |
| EMX2OS | hsa-miR-1721 |
| EMX2OS | hsa-miR-4770 |
| EMX2OS | hsa-miR-148ab-3p |
| EMX2OS | hsa-miR-152 |
| EMX2OS | hsa-miR-150 |
| EMX2OS | hsa-miR-5127 |
| EMX2OS | hsa-miR-182 |
| EMX2OS | hsa-miR-183 |
| EMX2OS | hsa-miR-184 |
| EMX2OS | hsa-miR-205 |
| EMX2OS | hsa-miR-205ab |
| EMX2OS | hsa-miR-210 |
| EMX2OS | hsa-miR-216a |
| EMX2OS | hsa-miR-22 |
| EMX2OS | hsa-miR-22-3p |
| EMX2OS | hsa-miR-24 |
| EMX2OS | hsa-miR-24ab |
| EMX2OS | hsa-miR-24-3p |
| EMX2OS | hsa-miR-31 |
| EMX2OS | hsa-miR-124 |
| EMX2OS | hsa-miR-124ab |
| EMX2OS | hsa-miR-506 |
| EMX2OS | hsa-miR-34ac |
| EMX2OS | hsa-miR-34bc-5p |
| EMX2OS | hsa-miR-449abc |
| EMX2OS | hsa-miR-449c-5p |
| EMX2OS | hsa-miR-455-5p |
| KIF25-AS1 | hsa-miR-132 |
| KIF25-AS1 | hsa-miR-212 |
| KIF25-AS1 | hsa-miR-212-3p |
| KIF25-AS1 | hsa-miR-7 |
| KIF25-AS1 | hsa-miR-7ab |
| KIF25-AS1 | hsa-miR-9 |
| KIF25-AS1 | hsa-miR-9ab |
| KIF25-AS1 | hsa-miR-138 |
| KIF25-AS1 | hsa-miR-138ab |
| KIF25-AS1 | hsa-miR-142-3p |
| KIF25-AS1 | hsa-miR-145 |
| KIF25-AS1 | hsa-miR-148ab-3p |
| KIF25-AS1 | hsa-miR-152 |
| KIF25-AS1 | hsa-miR-150 |
| KIF25-AS1 | hsa-miR-5127 |
| KIF25-AS1 | hsa-miR-15abc |
| KIF25-AS1 | hsa-miR-16 |
| KIF25-AS1 | hsa-miR-16abc |
| KIF25-AS1 | hsa-miR-195 |
| KIF25-AS1 | hsa-miR-322 |
| KIF25-AS1 | hsa-miR-424 |
| KIF25-AS1 | hsa-miR-497 |
| KIF25-AS1 | hsa-miR-1907 |
| KIF25-AS1 | hsa-miR-181abcd |
| KIF25-AS1 | hsa-miR-4262 |
| KIF25-AS1 | hsa-miR-192 |
| KIF25-AS1 | hsa-miR-215 |
| KIF25-AS1 | hsa-miR-193 |
| KIF25-AS1 | hsa-miR-193b |
| KIF25-AS1 | hsa-miR-193a-3p |
| KIF25-AS1 | hsa-miR-196abc |
| KIF25-AS1 | hsa-miR-19ab |
| KIF25-AS1 | hsa-miR-204 |
| KIF25-AS1 | hsa-miR-204b |
| KIF25-AS1 | hsa-miR-211 |
| KIF25-AS1 | hsa-miR-214 |
| KIF25-AS1 | hsa-miR-761 |
| KIF25-AS1 | hsa-miR-3619-5p |
| KIF25-AS1 | hsa-miR-218 |
| KIF25-AS1 | hsa-miR-218a |
| KIF25-AS1 | hsa-miR-24 |
| KIF25-AS1 | hsa-miR-24ab |
| KIF25-AS1 | hsa-miR-24-3p |
| KIF25-AS1 | hsa-miR-25 |
| KIF25-AS1 | hsa-miR-32 |
| KIF25-AS1 | hsa-miR-92abc |
| KIF25-AS1 | hsa-miR-363 |
| KIF25-AS1 | hsa-miR-363-3p |
| KIF25-AS1 | hsa-miR-367 |
| KIF25-AS1 | hsa-miR-27abc |
| KIF25-AS1 | hsa-miR-27a-3p |
| KIF25-AS1 | hsa-miR-103a |
| KIF25-AS1 | hsa-miR-107 |
| KIF25-AS1 | hsa-miR-107ab |
| KIF25-AS1 | hsa-miR-338 |
| KIF25-AS1 | hsa-miR-338-3p |
| KIF25-AS1 | hsa-miR-129-5p |
| KIF25-AS1 | hsa-miR-129ab-5p |
| MIR497HG | hsa-miR-18ab |
| MIR497HG | hsa-miR-4735-3p |
| MIR497HG | hsa-miR-23abc |
| MIR497HG | hsa-miR-23b-3p |
| MIR497HG | hsa-miR-24 |
| MIR497HG | hsa-miR-24ab |
| MIR497HG | hsa-miR-24-3p |
| MIR497HG | hsa-miR-125a-5p |
| MIR497HG | hsa-miR-125b-5p |
| MIR497HG | hsa-miR-351 |
| MIR497HG | hsa-miR-670 |
| MIR497HG | hsa-miR-4319 |
| LINC00028 | hsa-miR-7 |
| LINC00028 | hsa-miR-7ab |
| LINC00028 | hsa-miR-150 |
| LINC00028 | hsa-miR-5127 |
| LINC00028 | hsa-miR-15abc |
| LINC00028 | hsa-miR-16 |
| LINC00028 | hsa-miR-16abc |
| LINC00028 | hsa-miR-195 |
| LINC00028 | hsa-miR-322 |
| LINC00028 | hsa-miR-424 |
| LINC00028 | hsa-miR-497 |
| LINC00028 | hsa-miR-1907 |
| LINC00028 | hsa-miR-204 |
| LINC00028 | hsa-miR-204b |
| LINC00028 | hsa-miR-211 |
| LINC00028 | hsa-miR-216a |
| LINC00028 | hsa-miR-24 |
| LINC00028 | hsa-miR-24ab |
| LINC00028 | hsa-miR-24-3p |
| LINC00028 | hsa-miR-27abc |
| LINC00028 | hsa-miR-27a-3p |
| LINC00028 | hsa-miR-383 |
| TPRG1-AS1 | hsa-miR-133abc |
| TPRG1-AS1 | hsa-miR-9 |
| TPRG1-AS1 | hsa-miR-9ab |
| TPRG1-AS1 | hsa-miR-93 |
| TPRG1-AS1 | hsa-miR-93a |
| TPRG1-AS1 | hsa-miR-105 |
| TPRG1-AS1 | hsa-miR-106a |
| TPRG1-AS1 | hsa-miR-291a-3p |
| TPRG1-AS1 | hsa-miR-294 |
| TPRG1-AS1 | hsa-miR-295 |
| TPRG1-AS1 | hsa-miR-302abcde |
| TPRG1-AS1 | hsa-miR-372 |
| TPRG1-AS1 | hsa-miR-373 |
| TPRG1-AS1 | hsa-miR-428 |
| TPRG1-AS1 | hsa-miR-519a |
| TPRG1-AS1 | hsa-miR-520be |
| TPRG1-AS1 | hsa-miR-520acd-3p |
| TPRG1-AS1 | hsa-miR-1378 |
| TPRG1-AS1 | hsa-miR-1420ac |
| TPRG1-AS1 | hsa-miR-135ab |
| TPRG1-AS1 | hsa-miR-135a-5p |
| TPRG1-AS1 | hsa-miR-150 |
| TPRG1-AS1 | hsa-miR-5127 |
| TPRG1-AS1 | hsa-miR-17 |
| TPRG1-AS1 | hsa-miR-17-5p |
| TPRG1-AS1 | hsa-miR-20ab |
| TPRG1-AS1 | hsa-miR-20b-5p |
| TPRG1-AS1 | hsa-miR-106ab |
| TPRG1-AS1 | hsa-miR-427 |
| TPRG1-AS1 | hsa-miR-518a-3p |
| TPRG1-AS1 | hsa-miR-519d |
| TPRG1-AS1 | hsa-miR-182 |
| TPRG1-AS1 | hsa-miR-let-7 |
| TPRG1-AS1 | hsa-miR-98 |
| TPRG1-AS1 | hsa-miR-4458 |
| TPRG1-AS1 | hsa-miR-4500 |
| TPRG1-AS1 | hsa-miR-210 |
| TPRG1-AS1 | hsa-miR-25 |
| TPRG1-AS1 | hsa-miR-32 |
| TPRG1-AS1 | hsa-miR-92abc |
| TPRG1-AS1 | hsa-miR-363 |
| TPRG1-AS1 | hsa-miR-363-3p |
| TPRG1-AS1 | hsa-miR-367 |
| TPRG1-AS1 | hsa-miR-29abcd |
| TPRG1-AS1 | hsa-miR-338 |
| TPRG1-AS1 | hsa-miR-338-3p |
| TPRG1-AS1 | hsa-miR-455-5p |
| MAGI2-AS3 | hsa-miR-503 |
| MAGI2-AS3 | hsa-miR-132 |
| MAGI2-AS3 | hsa-miR-212 |
| MAGI2-AS3 | hsa-miR-212-3p |
| MAGI2-AS3 | hsa-miR-133abc |
| MAGI2-AS3 | hsa-miR-93 |
| MAGI2-AS3 | hsa-miR-93a |
| MAGI2-AS3 | hsa-miR-105 |
| MAGI2-AS3 | hsa-miR-106a |
| MAGI2-AS3 | hsa-miR-291a-3p |
| MAGI2-AS3 | hsa-miR-294 |
| MAGI2-AS3 | hsa-miR-295 |
| MAGI2-AS3 | hsa-miR-302abcde |
| MAGI2-AS3 | hsa-miR-372 |
| MAGI2-AS3 | hsa-miR-373 |
| MAGI2-AS3 | hsa-miR-428 |
| MAGI2-AS3 | hsa-miR-519a |
| MAGI2-AS3 | hsa-miR-520be |
| MAGI2-AS3 | hsa-miR-520acd-3p |
| MAGI2-AS3 | hsa-miR-1378 |
| MAGI2-AS3 | hsa-miR-1420ac |
| MAGI2-AS3 | hsa-miR-137 |
| MAGI2-AS3 | hsa-miR-137ab |
| MAGI2-AS3 | hsa-miR-138 |
| MAGI2-AS3 | hsa-miR-138ab |
| MAGI2-AS3 | hsa-miR-141 |
| MAGI2-AS3 | hsa-miR-200a |
| MAGI2-AS3 | hsa-miR-142-3p |
| MAGI2-AS3 | hsa-miR-143 |
| MAGI2-AS3 | hsa-miR-1721 |
| MAGI2-AS3 | hsa-miR-4770 |
| MAGI2-AS3 | hsa-miR-144 |
| MAGI2-AS3 | hsa-miR-145 |
| MAGI2-AS3 | hsa-miR-148ab-3p |
| MAGI2-AS3 | hsa-miR-152 |
| MAGI2-AS3 | hsa-miR-153 |
| MAGI2-AS3 | hsa-miR-155 |
| MAGI2-AS3 | hsa-miR-15abc |
| MAGI2-AS3 | hsa-miR-16 |
| MAGI2-AS3 | hsa-miR-16abc |
| MAGI2-AS3 | hsa-miR-195 |
| MAGI2-AS3 | hsa-miR-322 |
| MAGI2-AS3 | hsa-miR-424 |
| MAGI2-AS3 | hsa-miR-497 |
| MAGI2-AS3 | hsa-miR-1907 |
| MAGI2-AS3 | hsa-miR-181abcd |
| MAGI2-AS3 | hsa-miR-4262 |
| MAGI2-AS3 | hsa-miR-let-7 |
| MAGI2-AS3 | hsa-miR-98 |
| MAGI2-AS3 | hsa-miR-4458 |
| MAGI2-AS3 | hsa-miR-4500 |
| MAGI2-AS3 | hsa-miR-190 |
| MAGI2-AS3 | hsa-miR-190ab |
| MAGI2-AS3 | hsa-miR-194 |
| MAGI2-AS3 | hsa-miR-200bc |
| MAGI2-AS3 | hsa-miR-429 |
| MAGI2-AS3 | hsa-miR-548a |
| MAGI2-AS3 | hsa-miR-203 |
| MAGI2-AS3 | hsa-miR-204 |
| MAGI2-AS3 | hsa-miR-204b |
| MAGI2-AS3 | hsa-miR-211 |
| MAGI2-AS3 | hsa-miR-210 |
| MAGI2-AS3 | hsa-miR-214 |
| MAGI2-AS3 | hsa-miR-761 |
| MAGI2-AS3 | hsa-miR-3619-5p |
| MAGI2-AS3 | hsa-miR-216a |
| MAGI2-AS3 | hsa-miR-216b |
| MAGI2-AS3 | hsa-miR-216b-5p |
| MAGI2-AS3 | hsa-miR-217 |
| MAGI2-AS3 | hsa-miR-218 |
| MAGI2-AS3 | hsa-miR-218a |
| MAGI2-AS3 | hsa-miR-219-5p |
| MAGI2-AS3 | hsa-miR-508 |
| MAGI2-AS3 | hsa-miR-508-3p |
| MAGI2-AS3 | hsa-miR-4782-3p |
| MAGI2-AS3 | hsa-miR-22 |
| MAGI2-AS3 | hsa-miR-22-3p |
| MAGI2-AS3 | hsa-miR-223 |
| MAGI2-AS3 | hsa-miR-122 |
| MAGI2-AS3 | hsa-miR-122a |
| MAGI2-AS3 | hsa-miR-1352 |
| MAGI2-AS3 | hsa-miR-23abc |
| MAGI2-AS3 | hsa-miR-23b-3p |
| MAGI2-AS3 | hsa-miR-25 |
| MAGI2-AS3 | hsa-miR-32 |
| MAGI2-AS3 | hsa-miR-92abc |
| MAGI2-AS3 | hsa-miR-363 |
| MAGI2-AS3 | hsa-miR-363-3p |
| MAGI2-AS3 | hsa-miR-367 |
| MAGI2-AS3 | hsa-miR-27abc |
| MAGI2-AS3 | hsa-miR-27a-3p |
| MAGI2-AS3 | hsa-miR-31 |
| MAGI2-AS3 | hsa-miR-33a-3p |
| MAGI2-AS3 | hsa-miR-365 |
| MAGI2-AS3 | hsa-miR-365-3p |
| MAGI2-AS3 | hsa-miR-33ab |
| MAGI2-AS3 | hsa-miR-33-5p |
| MAGI2-AS3 | hsa-miR-425 |
| MAGI2-AS3 | hsa-miR-425-5p |
| MAGI2-AS3 | hsa-miR-489 |
| MAGI2-AS3 | hsa-miR-455-5p |
| MAGI2-AS3 | hsa-miR-128 |
| MAGI2-AS3 | hsa-miR-128ab |
| MAGI2-AS3 | hsa-miR-129-5p |
| MAGI2-AS3 | hsa-miR-129ab-5p |
| MAGI2-AS3 | hsa-miR-490-3p |
| MAGI2-AS3 | hsa-miR-499-5p |
| FNDC1-IT1 | hsa-miR-144 |
| FNDC1-IT1 | hsa-miR-153 |
| FNDC1-IT1 | hsa-miR-216b |
| FNDC1-IT1 | hsa-miR-216b-5p |
| FNDC1-IT1 | hsa-miR-221 |
| FNDC1-IT1 | hsa-miR-222 |
| FNDC1-IT1 | hsa-miR-222ab |
| FNDC1-IT1 | hsa-miR-1928 |
| FNDC1-IT1 | hsa-miR-23abc |
| FNDC1-IT1 | hsa-miR-23b-3p |
| FNDC1-IT1 | hsa-miR-24 |
| FNDC1-IT1 | hsa-miR-24ab |
| FNDC1-IT1 | hsa-miR-24-3p |
| FNDC1-IT1 | hsa-miR-101 |
| FNDC1-IT1 | hsa-miR-101ab |
| FNDC1-IT1 | hsa-miR-129-5p |
| FNDC1-IT1 | hsa-miR-129ab-5p |
| LINC00484 | hsa-miR-551a |
| LINC00484 | hsa-miR-133abc |
| LINC00484 | hsa-miR-9 |
| LINC00484 | hsa-miR-9ab |
| LINC00484 | hsa-miR-93 |
| LINC00484 | hsa-miR-93a |
| LINC00484 | hsa-miR-105 |
| LINC00484 | hsa-miR-106a |
| LINC00484 | hsa-miR-291a-3p |
| LINC00484 | hsa-miR-294 |
| LINC00484 | hsa-miR-295 |
| LINC00484 | hsa-miR-302abcde |
| LINC00484 | hsa-miR-372 |
| LINC00484 | hsa-miR-373 |
| LINC00484 | hsa-miR-428 |
| LINC00484 | hsa-miR-519a |
| LINC00484 | hsa-miR-520be |
| LINC00484 | hsa-miR-520acd-3p |
| LINC00484 | hsa-miR-1378 |
| LINC00484 | hsa-miR-1420ac |
| LINC00484 | hsa-miR-138 |
| LINC00484 | hsa-miR-138ab |
| LINC00484 | hsa-miR-139-5p |
| LINC00484 | hsa-miR-141 |
| LINC00484 | hsa-miR-200a |
| LINC00484 | hsa-miR-142-3p |
| LINC00484 | hsa-miR-143 |
| LINC00484 | hsa-miR-1721 |
| LINC00484 | hsa-miR-4770 |
| LINC00484 | hsa-miR-148ab-3p |
| LINC00484 | hsa-miR-152 |
| LINC00484 | hsa-miR-15abc |
| LINC00484 | hsa-miR-16 |
| LINC00484 | hsa-miR-16abc |
| LINC00484 | hsa-miR-195 |
| LINC00484 | hsa-miR-322 |
| LINC00484 | hsa-miR-424 |
| LINC00484 | hsa-miR-497 |
| LINC00484 | hsa-miR-1907 |
| LINC00484 | hsa-miR-let-7 |
| LINC00484 | hsa-miR-98 |
| LINC00484 | hsa-miR-4458 |
| LINC00484 | hsa-miR-4500 |
| LINC00484 | hsa-miR-187 |
| LINC00484 | hsa-miR-19ab |
| LINC00484 | hsa-miR-1ab |
| LINC00484 | hsa-miR-206 |
| LINC00484 | hsa-miR-613 |
| LINC00484 | hsa-miR-214 |
| LINC00484 | hsa-miR-761 |
| LINC00484 | hsa-miR-3619-5p |
| LINC00484 | hsa-miR-217 |
| LINC00484 | hsa-miR-218 |
| LINC00484 | hsa-miR-218a |
| LINC00484 | hsa-miR-22 |
| LINC00484 | hsa-miR-22-3p |
| LINC00484 | hsa-miR-223 |
| LINC00484 | hsa-miR-122 |
| LINC00484 | hsa-miR-122a |
| LINC00484 | hsa-miR-1352 |
| LINC00484 | hsa-miR-25 |
| LINC00484 | hsa-miR-32 |
| LINC00484 | hsa-miR-92abc |
| LINC00484 | hsa-miR-363 |
| LINC00484 | hsa-miR-363-3p |
| LINC00484 | hsa-miR-367 |
| LINC00484 | hsa-miR-26ab |
| LINC00484 | hsa-miR-1297 |
| LINC00484 | hsa-miR-4465 |
| LINC00484 | hsa-miR-29abcd |
| LINC00484 | hsa-miR-103a |
| LINC00484 | hsa-miR-107 |
| LINC00484 | hsa-miR-107ab |
| LINC00484 | hsa-miR-124 |
| LINC00484 | hsa-miR-124ab |
| LINC00484 | hsa-miR-506 |
| LINC00484 | hsa-miR-338 |
| LINC00484 | hsa-miR-338-3p |
| LINC00484 | hsa-miR-33ab |
| LINC00484 | hsa-miR-33-5p |
| LINC00484 | hsa-miR-34ac |
| LINC00484 | hsa-miR-34bc-5p |
| LINC00484 | hsa-miR-449abc |
| LINC00484 | hsa-miR-449c-5p |
| LINC00484 | hsa-miR-455-5p |
| LINC00484 | hsa-miR-490-3p |
| DIAPH2-AS1 | hsa-miR-9 |
| DIAPH2-AS1 | hsa-miR-9ab |
| DIAPH2-AS1 | hsa-miR-140 |
| DIAPH2-AS1 | hsa-miR-140-5p |
| DIAPH2-AS1 | hsa-miR-876-3p |
| DIAPH2-AS1 | hsa-miR-1244 |
| DIAPH2-AS1 | hsa-miR-153 |
| DIAPH2-AS1 | hsa-miR-181abcd |
| DIAPH2-AS1 | hsa-miR-4262 |
| DIAPH2-AS1 | hsa-miR-183 |
| DIAPH2-AS1 | hsa-miR-18ab |
| DIAPH2-AS1 | hsa-miR-4735-3p |
| DIAPH2-AS1 | hsa-miR-190 |
| DIAPH2-AS1 | hsa-miR-190ab |
| DIAPH2-AS1 | hsa-miR-200bc |
| DIAPH2-AS1 | hsa-miR-429 |
| DIAPH2-AS1 | hsa-miR-548a |
| DIAPH2-AS1 | hsa-miR-204 |
| DIAPH2-AS1 | hsa-miR-204b |
| DIAPH2-AS1 | hsa-miR-211 |
| DIAPH2-AS1 | hsa-miR-217 |
| DIAPH2-AS1 | hsa-miR-122 |
| DIAPH2-AS1 | hsa-miR-122a |
| DIAPH2-AS1 | hsa-miR-1352 |
| DIAPH2-AS1 | hsa-miR-23abc |
| DIAPH2-AS1 | hsa-miR-23b-3p |
| DIAPH2-AS1 | hsa-miR-34ac |
| DIAPH2-AS1 | hsa-miR-34bc-5p |
| DIAPH2-AS1 | hsa-miR-449abc |
| DIAPH2-AS1 | hsa-miR-449c-5p |
| DIAPH2-AS1 | hsa-miR-425 |
| DIAPH2-AS1 | hsa-miR-425-5p |
| DIAPH2-AS1 | hsa-miR-489 |
| DIAPH2-AS1 | hsa-miR-129-5p |
| DIAPH2-AS1 | hsa-miR-129ab-5p |
| DGUOK-AS1 | hsa-miR-135ab |
| DGUOK-AS1 | hsa-miR-135a-5p |
| DGUOK-AS1 | hsa-miR-138 |
| DGUOK-AS1 | hsa-miR-138ab |
| DGUOK-AS1 | hsa-miR-145 |
| DGUOK-AS1 | hsa-miR-15abc |
| DGUOK-AS1 | hsa-miR-16 |
| DGUOK-AS1 | hsa-miR-16abc |
| DGUOK-AS1 | hsa-miR-195 |
| DGUOK-AS1 | hsa-miR-322 |
| DGUOK-AS1 | hsa-miR-424 |
| DGUOK-AS1 | hsa-miR-497 |
| DGUOK-AS1 | hsa-miR-1907 |
| DGUOK-AS1 | hsa-miR-199ab-5p |
| DGUOK-AS1 | hsa-miR-204 |
| DGUOK-AS1 | hsa-miR-204b |
| DGUOK-AS1 | hsa-miR-211 |
| DGUOK-AS1 | hsa-miR-214 |
| DGUOK-AS1 | hsa-miR-761 |
| DGUOK-AS1 | hsa-miR-3619-5p |
| DGUOK-AS1 | hsa-miR-223 |
| DGUOK-AS1 | hsa-miR-103a |
| DGUOK-AS1 | hsa-miR-107 |
| DGUOK-AS1 | hsa-miR-107ab |
| DGUOK-AS1 | hsa-miR-33a-3p |
| DGUOK-AS1 | hsa-miR-365 |
| DGUOK-AS1 | hsa-miR-365-3p |
| DGUOK-AS1 | hsa-miR-499-5p |
| BRWD1-AS1 | hsa-miR-9 |
| BRWD1-AS1 | hsa-miR-9ab |
| BRWD1-AS1 | hsa-miR-137 |
| BRWD1-AS1 | hsa-miR-137ab |
| BRWD1-AS1 | hsa-miR-153 |
| BRWD1-AS1 | hsa-miR-216a |
| BRWD1-AS1 | hsa-miR-216b |
| BRWD1-AS1 | hsa-miR-216b-5p |
| BRWD1-AS1 | hsa-miR-24 |
| BRWD1-AS1 | hsa-miR-24ab |
| BRWD1-AS1 | hsa-miR-24-3p |
| BRWD1-AS1 | hsa-miR-375 |
| ADAMTS9-AS1 | hsa-miR-130ac |
| ADAMTS9-AS1 | hsa-miR-301ab |
| ADAMTS9-AS1 | hsa-miR-301b |
| ADAMTS9-AS1 | hsa-miR-301b-3p |
| ADAMTS9-AS1 | hsa-miR-454 |
| ADAMTS9-AS1 | hsa-miR-721 |
| ADAMTS9-AS1 | hsa-miR-4295 |
| ADAMTS9-AS1 | hsa-miR-3666 |
| ADAMTS9-AS1 | hsa-miR-96 |
| ADAMTS9-AS1 | hsa-miR-507 |
| ADAMTS9-AS1 | hsa-miR-1271 |
| ADAMTS9-AS1 | hsa-miR-144 |
| ADAMTS9-AS1 | hsa-miR-145 |
| ADAMTS9-AS1 | hsa-miR-146ac |
| ADAMTS9-AS1 | hsa-miR-146b-5p |
| ADAMTS9-AS1 | hsa-miR-150 |
| ADAMTS9-AS1 | hsa-miR-5127 |
| ADAMTS9-AS1 | hsa-miR-155 |
| ADAMTS9-AS1 | hsa-miR-181abcd |
| ADAMTS9-AS1 | hsa-miR-4262 |
| ADAMTS9-AS1 | hsa-miR-182 |
| ADAMTS9-AS1 | hsa-miR-199ab-5p |
| ADAMTS9-AS1 | hsa-miR-21 |
| ADAMTS9-AS1 | hsa-miR-590-5p |
| ADAMTS9-AS1 | hsa-miR-214 |
| ADAMTS9-AS1 | hsa-miR-761 |
| ADAMTS9-AS1 | hsa-miR-3619-5p |
| ADAMTS9-AS1 | hsa-miR-216a |
| ADAMTS9-AS1 | hsa-miR-26ab |
| ADAMTS9-AS1 | hsa-miR-1297 |
| ADAMTS9-AS1 | hsa-miR-4465 |
| ADAMTS9-AS1 | hsa-miR-27abc |
| ADAMTS9-AS1 | hsa-miR-27a-3p |
| ADAMTS9-AS1 | hsa-miR-101 |
| ADAMTS9-AS1 | hsa-miR-101ab |
| ADAMTS9-AS1 | hsa-miR-29abcd |
| ADAMTS9-AS1 | hsa-miR-31 |
| ADAMTS9-AS1 | hsa-miR-455-5p |
| ADAMTS9-AS1 | hsa-miR-128 |
| ADAMTS9-AS1 | hsa-miR-128ab |
| ADAMTS9-AS2 | hsa-miR-130ac |
| ADAMTS9-AS2 | hsa-miR-301ab |
| ADAMTS9-AS2 | hsa-miR-301b |
| ADAMTS9-AS2 | hsa-miR-301b-3p |
| ADAMTS9-AS2 | hsa-miR-454 |
| ADAMTS9-AS2 | hsa-miR-721 |
| ADAMTS9-AS2 | hsa-miR-4295 |
| ADAMTS9-AS2 | hsa-miR-3666 |
| ADAMTS9-AS2 | hsa-miR-132 |
| ADAMTS9-AS2 | hsa-miR-212 |
| ADAMTS9-AS2 | hsa-miR-212-3p |
| ADAMTS9-AS2 | hsa-miR-7 |
| ADAMTS9-AS2 | hsa-miR-7ab |
| ADAMTS9-AS2 | hsa-miR-93 |
| ADAMTS9-AS2 | hsa-miR-93a |
| ADAMTS9-AS2 | hsa-miR-105 |
| ADAMTS9-AS2 | hsa-miR-106a |
| ADAMTS9-AS2 | hsa-miR-291a-3p |
| ADAMTS9-AS2 | hsa-miR-294 |
| ADAMTS9-AS2 | hsa-miR-295 |
| ADAMTS9-AS2 | hsa-miR-302abcde |
| ADAMTS9-AS2 | hsa-miR-372 |
| ADAMTS9-AS2 | hsa-miR-373 |
| ADAMTS9-AS2 | hsa-miR-428 |
| ADAMTS9-AS2 | hsa-miR-519a |
| ADAMTS9-AS2 | hsa-miR-520be |
| ADAMTS9-AS2 | hsa-miR-520acd-3p |
| ADAMTS9-AS2 | hsa-miR-1378 |
| ADAMTS9-AS2 | hsa-miR-1420ac |
| ADAMTS9-AS2 | hsa-miR-96 |
| ADAMTS9-AS2 | hsa-miR-507 |
| ADAMTS9-AS2 | hsa-miR-1271 |
| ADAMTS9-AS2 | hsa-miR-135ab |
| ADAMTS9-AS2 | hsa-miR-135a-5p |
| ADAMTS9-AS2 | hsa-miR-137 |
| ADAMTS9-AS2 | hsa-miR-137ab |
| ADAMTS9-AS2 | hsa-miR-140 |
| ADAMTS9-AS2 | hsa-miR-140-5p |
| ADAMTS9-AS2 | hsa-miR-876-3p |
| ADAMTS9-AS2 | hsa-miR-1244 |
| ADAMTS9-AS2 | hsa-miR-141 |
| ADAMTS9-AS2 | hsa-miR-200a |
| ADAMTS9-AS2 | hsa-miR-143 |
| ADAMTS9-AS2 | hsa-miR-1721 |
| ADAMTS9-AS2 | hsa-miR-4770 |
| ADAMTS9-AS2 | hsa-miR-144 |
| ADAMTS9-AS2 | hsa-miR-145 |
| ADAMTS9-AS2 | hsa-miR-148ab-3p |
| ADAMTS9-AS2 | hsa-miR-152 |
| ADAMTS9-AS2 | hsa-miR-150 |
| ADAMTS9-AS2 | hsa-miR-5127 |
| ADAMTS9-AS2 | hsa-miR-153 |
| ADAMTS9-AS2 | hsa-miR-155 |
| ADAMTS9-AS2 | hsa-miR-181abcd |
| ADAMTS9-AS2 | hsa-miR-4262 |
| ADAMTS9-AS2 | hsa-miR-182 |
| ADAMTS9-AS2 | hsa-miR-183 |
| ADAMTS9-AS2 | hsa-miR-184 |
| ADAMTS9-AS2 | hsa-miR-let-7 |
| ADAMTS9-AS2 | hsa-miR-98 |
| ADAMTS9-AS2 | hsa-miR-4458 |
| ADAMTS9-AS2 | hsa-miR-4500 |
| ADAMTS9-AS2 | hsa-miR-18ab |
| ADAMTS9-AS2 | hsa-miR-4735-3p |
| ADAMTS9-AS2 | hsa-miR-190 |
| ADAMTS9-AS2 | hsa-miR-190ab |
| ADAMTS9-AS2 | hsa-miR-193 |
| ADAMTS9-AS2 | hsa-miR-193b |
| ADAMTS9-AS2 | hsa-miR-193a-3p |
| ADAMTS9-AS2 | hsa-miR-196abc |
| ADAMTS9-AS2 | hsa-miR-199ab-5p |
| ADAMTS9-AS2 | hsa-miR-19ab |
| ADAMTS9-AS2 | hsa-miR-203 |
| ADAMTS9-AS2 | hsa-miR-204 |
| ADAMTS9-AS2 | hsa-miR-204b |
| ADAMTS9-AS2 | hsa-miR-211 |
| ADAMTS9-AS2 | hsa-miR-205 |
| ADAMTS9-AS2 | hsa-miR-205ab |
| ADAMTS9-AS2 | hsa-miR-208ab |
| ADAMTS9-AS2 | hsa-miR-208ab-3p |
| ADAMTS9-AS2 | hsa-miR-214 |
| ADAMTS9-AS2 | hsa-miR-761 |
| ADAMTS9-AS2 | hsa-miR-3619-5p |
| ADAMTS9-AS2 | hsa-miR-216a |
| ADAMTS9-AS2 | hsa-miR-216b |
| ADAMTS9-AS2 | hsa-miR-216b-5p |
| ADAMTS9-AS2 | hsa-miR-218 |
| ADAMTS9-AS2 | hsa-miR-218a |
| ADAMTS9-AS2 | hsa-miR-22 |
| ADAMTS9-AS2 | hsa-miR-22-3p |
| ADAMTS9-AS2 | hsa-miR-221 |
| ADAMTS9-AS2 | hsa-miR-222 |
| ADAMTS9-AS2 | hsa-miR-222ab |
| ADAMTS9-AS2 | hsa-miR-1928 |
| ADAMTS9-AS2 | hsa-miR-223 |
| ADAMTS9-AS2 | hsa-miR-122 |
| ADAMTS9-AS2 | hsa-miR-122a |
| ADAMTS9-AS2 | hsa-miR-1352 |
| ADAMTS9-AS2 | hsa-miR-23abc |
| ADAMTS9-AS2 | hsa-miR-23b-3p |
| ADAMTS9-AS2 | hsa-miR-24 |
| ADAMTS9-AS2 | hsa-miR-24ab |
| ADAMTS9-AS2 | hsa-miR-24-3p |
| ADAMTS9-AS2 | hsa-miR-25 |
| ADAMTS9-AS2 | hsa-miR-32 |
| ADAMTS9-AS2 | hsa-miR-92abc |
| ADAMTS9-AS2 | hsa-miR-363 |
| ADAMTS9-AS2 | hsa-miR-363-3p |
| ADAMTS9-AS2 | hsa-miR-367 |
| ADAMTS9-AS2 | hsa-miR-26ab |
| ADAMTS9-AS2 | hsa-miR-1297 |
| ADAMTS9-AS2 | hsa-miR-4465 |
| ADAMTS9-AS2 | hsa-miR-27abc |
| ADAMTS9-AS2 | hsa-miR-27a-3p |
| ADAMTS9-AS2 | hsa-miR-101 |
| ADAMTS9-AS2 | hsa-miR-101ab |
| ADAMTS9-AS2 | hsa-miR-30abcdef |
| ADAMTS9-AS2 | hsa-miR-30abe-5p |
| ADAMTS9-AS2 | hsa-miR-384-5p |
| ADAMTS9-AS2 | hsa-miR-31 |
| ADAMTS9-AS2 | hsa-miR-103a |
| ADAMTS9-AS2 | hsa-miR-107 |
| ADAMTS9-AS2 | hsa-miR-107ab |
| ADAMTS9-AS2 | hsa-miR-338 |
| ADAMTS9-AS2 | hsa-miR-338-3p |
| ADAMTS9-AS2 | hsa-miR-33a-3p |
| ADAMTS9-AS2 | hsa-miR-365 |
| ADAMTS9-AS2 | hsa-miR-365-3p |
| ADAMTS9-AS2 | hsa-miR-33ab |
| ADAMTS9-AS2 | hsa-miR-33-5p |
| ADAMTS9-AS2 | hsa-miR-34ac |
| ADAMTS9-AS2 | hsa-miR-34bc-5p |
| ADAMTS9-AS2 | hsa-miR-449abc |
| ADAMTS9-AS2 | hsa-miR-449c-5p |
| ADAMTS9-AS2 | hsa-miR-375 |
| ADAMTS9-AS2 | hsa-miR-10abc |
| ADAMTS9-AS2 | hsa-miR-10a-5p |
| ADAMTS9-AS2 | hsa-miR-451 |
| ADAMTS9-AS2 | hsa-miR-128 |
| ADAMTS9-AS2 | hsa-miR-128ab |
| ADAMTS9-AS2 | hsa-miR-129-5p |
| ADAMTS9-AS2 | hsa-miR-129ab-5p |
| ADAMTS9-AS2 | hsa-miR-499-5p |
| SNHG3 | hsa-miR-93 |
| SNHG3 | hsa-miR-93a |
| SNHG3 | hsa-miR-105 |
| SNHG3 | hsa-miR-106a |
| SNHG3 | hsa-miR-291a-3p |
| SNHG3 | hsa-miR-294 |
| SNHG3 | hsa-miR-295 |
| SNHG3 | hsa-miR-302abcde |
| SNHG3 | hsa-miR-372 |
| SNHG3 | hsa-miR-373 |
| SNHG3 | hsa-miR-428 |
| SNHG3 | hsa-miR-519a |
| SNHG3 | hsa-miR-520be |
| SNHG3 | hsa-miR-520acd-3p |
| SNHG3 | hsa-miR-1378 |
| SNHG3 | hsa-miR-1420ac |
| SNHG3 | hsa-miR-135ab |
| SNHG3 | hsa-miR-135a-5p |
| SNHG3 | hsa-miR-139-5p |
| SNHG3 | hsa-miR-141 |
| SNHG3 | hsa-miR-200a |
| SNHG3 | hsa-miR-146ac |
| SNHG3 | hsa-miR-146b-5p |
| SNHG3 | hsa-miR-148ab-3p |
| SNHG3 | hsa-miR-152 |
| SNHG3 | hsa-miR-17 |
| SNHG3 | hsa-miR-17-5p |
| SNHG3 | hsa-miR-20ab |
| SNHG3 | hsa-miR-20b-5p |
| SNHG3 | hsa-miR-106ab |
| SNHG3 | hsa-miR-427 |
| SNHG3 | hsa-miR-518a-3p |
| SNHG3 | hsa-miR-519d |
| SNHG3 | hsa-miR-182 |
| SNHG3 | hsa-miR-196abc |
| SNHG3 | hsa-miR-19ab |
| SNHG3 | hsa-miR-1ab |
| SNHG3 | hsa-miR-206 |
| SNHG3 | hsa-miR-613 |
| SNHG3 | hsa-miR-203 |
| SNHG3 | hsa-miR-205 |
| SNHG3 | hsa-miR-205ab |
| SNHG3 | hsa-miR-208ab |
| SNHG3 | hsa-miR-208ab-3p |
| SNHG3 | hsa-miR-214 |
| SNHG3 | hsa-miR-761 |
| SNHG3 | hsa-miR-3619-5p |
| SNHG3 | hsa-miR-216a |
| SNHG3 | hsa-miR-219-5p |
| SNHG3 | hsa-miR-508 |
| SNHG3 | hsa-miR-508-3p |
| SNHG3 | hsa-miR-4782-3p |
| SNHG3 | hsa-miR-221 |
| SNHG3 | hsa-miR-222 |
| SNHG3 | hsa-miR-222ab |
| SNHG3 | hsa-miR-1928 |
| SNHG3 | hsa-miR-122 |
| SNHG3 | hsa-miR-122a |
| SNHG3 | hsa-miR-1352 |
| SNHG3 | hsa-miR-24 |
| SNHG3 | hsa-miR-24ab |
| SNHG3 | hsa-miR-24-3p |
| SNHG3 | hsa-miR-101 |
| SNHG3 | hsa-miR-101ab |
| SNHG3 | hsa-miR-31 |
| SNHG3 | hsa-miR-338 |
| SNHG3 | hsa-miR-338-3p |
| SNHG3 | hsa-miR-10abc |
| SNHG3 | hsa-miR-10a-5p |
| SNHG3 | hsa-miR-455-5p |
| SNHG3 | hsa-miR-128 |
| SNHG3 | hsa-miR-128ab |
| SNHG3 | hsa-miR-129-5p |
| SNHG3 | hsa-miR-129ab-5p |
| SNHG3 | hsa-miR-490-3p |
| SNHG3 | hsa-miR-499-5p |
| CRNDE | hsa-miR-9 |
| CRNDE | hsa-miR-9ab |
| CRNDE | hsa-miR-135ab |
| CRNDE | hsa-miR-135a-5p |
| CRNDE | hsa-miR-140 |
| CRNDE | hsa-miR-140-5p |
| CRNDE | hsa-miR-876-3p |
| CRNDE | hsa-miR-1244 |
| CRNDE | hsa-miR-142-3p |
| CRNDE | hsa-miR-143 |
| CRNDE | hsa-miR-1721 |
| CRNDE | hsa-miR-4770 |
| CRNDE | hsa-miR-144 |
| CRNDE | hsa-miR-145 |
| CRNDE | hsa-miR-146ac |
| CRNDE | hsa-miR-146b-5p |
| CRNDE | hsa-miR-155 |
| CRNDE | hsa-miR-181abcd |
| CRNDE | hsa-miR-4262 |
| CRNDE | hsa-miR-183 |
| CRNDE | hsa-miR-193 |
| CRNDE | hsa-miR-193b |
| CRNDE | hsa-miR-193a-3p |
| CRNDE | hsa-miR-199ab-5p |
| CRNDE | hsa-miR-203 |
| CRNDE | hsa-miR-205 |
| CRNDE | hsa-miR-205ab |
| CRNDE | hsa-miR-216b |
| CRNDE | hsa-miR-216b-5p |
| CRNDE | hsa-miR-217 |
| CRNDE | hsa-miR-219-5p |
| CRNDE | hsa-miR-508 |
| CRNDE | hsa-miR-508-3p |
| CRNDE | hsa-miR-4782-3p |
| CRNDE | hsa-miR-22 |
| CRNDE | hsa-miR-22-3p |
| CRNDE | hsa-miR-221 |
| CRNDE | hsa-miR-222 |
| CRNDE | hsa-miR-222ab |
| CRNDE | hsa-miR-1928 |
| CRNDE | hsa-miR-223 |
| CRNDE | hsa-miR-23abc |
| CRNDE | hsa-miR-23b-3p |
| CRNDE | hsa-miR-25 |
| CRNDE | hsa-miR-32 |
| CRNDE | hsa-miR-92abc |
| CRNDE | hsa-miR-363 |
| CRNDE | hsa-miR-363-3p |
| CRNDE | hsa-miR-367 |
| CRNDE | hsa-miR-26ab |
| CRNDE | hsa-miR-1297 |
| CRNDE | hsa-miR-4465 |
| CRNDE | hsa-miR-27abc |
| CRNDE | hsa-miR-27a-3p |
| CRNDE | hsa-miR-101 |
| CRNDE | hsa-miR-101ab |
| CRNDE | hsa-miR-31 |
| CRNDE | hsa-miR-338 |
| CRNDE | hsa-miR-338-3p |
| CRNDE | hsa-miR-128 |
| CRNDE | hsa-miR-128ab |
| CRNDE | hsa-miR-129-5p |
| CRNDE | hsa-miR-129ab-5p |
| PVT1 | hsa-miR-503 |
| PVT1 | hsa-miR-551a |
| PVT1 | hsa-miR-7 |
| PVT1 | hsa-miR-7ab |
| PVT1 | hsa-miR-133abc |
| PVT1 | hsa-miR-9 |
| PVT1 | hsa-miR-9ab |
| PVT1 | hsa-miR-93 |
| PVT1 | hsa-miR-93a |
| PVT1 | hsa-miR-105 |
| PVT1 | hsa-miR-106a |
| PVT1 | hsa-miR-291a-3p |
| PVT1 | hsa-miR-294 |
| PVT1 | hsa-miR-295 |
| PVT1 | hsa-miR-302abcde |
| PVT1 | hsa-miR-372 |
| PVT1 | hsa-miR-373 |
| PVT1 | hsa-miR-428 |
| PVT1 | hsa-miR-519a |
| PVT1 | hsa-miR-520be |
| PVT1 | hsa-miR-520acd-3p |
| PVT1 | hsa-miR-1378 |
| PVT1 | hsa-miR-1420ac |
| PVT1 | hsa-miR-139-5p |
| PVT1 | hsa-miR-140 |
| PVT1 | hsa-miR-140-5p |
| PVT1 | hsa-miR-876-3p |
| PVT1 | hsa-miR-1244 |
| PVT1 | hsa-miR-143 |
| PVT1 | hsa-miR-1721 |
| PVT1 | hsa-miR-4770 |
| PVT1 | hsa-miR-145 |
| PVT1 | hsa-miR-148ab-3p |
| PVT1 | hsa-miR-152 |
| PVT1 | hsa-miR-150 |
| PVT1 | hsa-miR-5127 |
| PVT1 | hsa-miR-15abc |
| PVT1 | hsa-miR-16 |
| PVT1 | hsa-miR-16abc |
| PVT1 | hsa-miR-195 |
| PVT1 | hsa-miR-322 |
| PVT1 | hsa-miR-424 |
| PVT1 | hsa-miR-497 |
| PVT1 | hsa-miR-1907 |
| PVT1 | hsa-miR-17 |
| PVT1 | hsa-miR-17-5p |
| PVT1 | hsa-miR-20ab |
| PVT1 | hsa-miR-20b-5p |
| PVT1 | hsa-miR-106ab |
| PVT1 | hsa-miR-427 |
| PVT1 | hsa-miR-518a-3p |
| PVT1 | hsa-miR-519d |
| PVT1 | hsa-miR-181abcd |
| PVT1 | hsa-miR-4262 |
| PVT1 | hsa-miR-183 |
| PVT1 | hsa-miR-187 |
| PVT1 | hsa-miR-18ab |
| PVT1 | hsa-miR-4735-3p |
| PVT1 | hsa-miR-190 |
| PVT1 | hsa-miR-190ab |
| PVT1 | hsa-miR-194 |
| PVT1 | hsa-miR-199ab-5p |
| PVT1 | hsa-miR-203 |
| PVT1 | hsa-miR-205 |
| PVT1 | hsa-miR-205ab |
| PVT1 | hsa-miR-21 |
| PVT1 | hsa-miR-590-5p |
| PVT1 | hsa-miR-214 |
| PVT1 | hsa-miR-761 |
| PVT1 | hsa-miR-3619-5p |
| PVT1 | hsa-miR-216a |
| PVT1 | hsa-miR-216b |
| PVT1 | hsa-miR-216b-5p |
| PVT1 | hsa-miR-217 |
| PVT1 | hsa-miR-221 |
| PVT1 | hsa-miR-222 |
| PVT1 | hsa-miR-222ab |
| PVT1 | hsa-miR-1928 |
| PVT1 | hsa-miR-23abc |
| PVT1 | hsa-miR-23b-3p |
| PVT1 | hsa-miR-24 |
| PVT1 | hsa-miR-24ab |
| PVT1 | hsa-miR-24-3p |
| PVT1 | hsa-miR-27abc |
| PVT1 | hsa-miR-27a-3p |
| PVT1 | hsa-miR-29abcd |
| PVT1 | hsa-miR-30abcdef |
| PVT1 | hsa-miR-30abe-5p |
| PVT1 | hsa-miR-384-5p |
| PVT1 | hsa-miR-31 |
| PVT1 | hsa-miR-124 |
| PVT1 | hsa-miR-124ab |
| PVT1 | hsa-miR-506 |
| PVT1 | hsa-miR-33a-3p |
| PVT1 | hsa-miR-365 |
| PVT1 | hsa-miR-365-3p |
| PVT1 | hsa-miR-34ac |
| PVT1 | hsa-miR-34bc-5p |
| PVT1 | hsa-miR-449abc |
| PVT1 | hsa-miR-449c-5p |
| PVT1 | hsa-miR-383 |
| PVT1 | hsa-miR-455-5p |
| PVT1 | hsa-miR-128 |
| PVT1 | hsa-miR-128ab |
| PVT1 | hsa-miR-490-3p |
| RRM1-AS1 | hsa-miR-145 |
| RRM1-AS1 | hsa-miR-184 |
| RRM1-AS1 | hsa-miR-187 |
| RRM1-AS1 | hsa-miR-1ab |
| RRM1-AS1 | hsa-miR-206 |
| RRM1-AS1 | hsa-miR-613 |
| SNHG1 | hsa-miR-503 |
| SNHG1 | hsa-miR-7 |
| SNHG1 | hsa-miR-7ab |
| SNHG1 | hsa-miR-9 |
| SNHG1 | hsa-miR-9ab |
| SNHG1 | hsa-miR-137 |
| SNHG1 | hsa-miR-137ab |
| SNHG1 | hsa-miR-140 |
| SNHG1 | hsa-miR-140-5p |
| SNHG1 | hsa-miR-876-3p |
| SNHG1 | hsa-miR-1244 |
| SNHG1 | hsa-miR-141 |
| SNHG1 | hsa-miR-200a |
| SNHG1 | hsa-miR-143 |
| SNHG1 | hsa-miR-1721 |
| SNHG1 | hsa-miR-4770 |
| SNHG1 | hsa-miR-144 |
| SNHG1 | hsa-miR-145 |
| SNHG1 | hsa-miR-146ac |
| SNHG1 | hsa-miR-146b-5p |
| SNHG1 | hsa-miR-153 |
| SNHG1 | hsa-miR-15abc |
| SNHG1 | hsa-miR-16 |
| SNHG1 | hsa-miR-16abc |
| SNHG1 | hsa-miR-195 |
| SNHG1 | hsa-miR-322 |
| SNHG1 | hsa-miR-424 |
| SNHG1 | hsa-miR-497 |
| SNHG1 | hsa-miR-1907 |
| SNHG1 | hsa-miR-181abcd |
| SNHG1 | hsa-miR-4262 |
| SNHG1 | hsa-miR-182 |
| SNHG1 | hsa-miR-18ab |
| SNHG1 | hsa-miR-4735-3p |
| SNHG1 | hsa-miR-194 |
| SNHG1 | hsa-miR-199ab-5p |
| SNHG1 | hsa-miR-1ab |
| SNHG1 | hsa-miR-206 |
| SNHG1 | hsa-miR-613 |
| SNHG1 | hsa-miR-204 |
| SNHG1 | hsa-miR-204b |
| SNHG1 | hsa-miR-211 |
| SNHG1 | hsa-miR-205 |
| SNHG1 | hsa-miR-205ab |
| SNHG1 | hsa-miR-208ab |
| SNHG1 | hsa-miR-208ab-3p |
| SNHG1 | hsa-miR-21 |
| SNHG1 | hsa-miR-590-5p |
| SNHG1 | hsa-miR-216b |
| SNHG1 | hsa-miR-216b-5p |
| SNHG1 | hsa-miR-217 |
| SNHG1 | hsa-miR-122 |
| SNHG1 | hsa-miR-122a |
| SNHG1 | hsa-miR-1352 |
| SNHG1 | hsa-miR-23abc |
| SNHG1 | hsa-miR-23b-3p |
| SNHG1 | hsa-miR-25 |
| SNHG1 | hsa-miR-32 |
| SNHG1 | hsa-miR-92abc |
| SNHG1 | hsa-miR-363 |
| SNHG1 | hsa-miR-363-3p |
| SNHG1 | hsa-miR-367 |
| SNHG1 | hsa-miR-101 |
| SNHG1 | hsa-miR-101ab |
| SNHG1 | hsa-miR-124 |
| SNHG1 | hsa-miR-124ab |
| SNHG1 | hsa-miR-506 |
| SNHG1 | hsa-miR-383 |
| SNHG1 | hsa-miR-128 |
| SNHG1 | hsa-miR-128ab |
| SNHG1 | hsa-miR-129-5p |
| SNHG1 | hsa-miR-129ab-5p |
| SNHG1 | hsa-miR-499-5p |
| DIO3OS | hsa-miR-7 |
| DIO3OS | hsa-miR-7ab |
| DIO3OS | hsa-miR-133abc |
| DIO3OS | hsa-miR-93 |
| DIO3OS | hsa-miR-93a |
| DIO3OS | hsa-miR-105 |
| DIO3OS | hsa-miR-106a |
| DIO3OS | hsa-miR-291a-3p |
| DIO3OS | hsa-miR-294 |
| DIO3OS | hsa-miR-295 |
| DIO3OS | hsa-miR-302abcde |
| DIO3OS | hsa-miR-372 |
| DIO3OS | hsa-miR-373 |
| DIO3OS | hsa-miR-428 |
| DIO3OS | hsa-miR-519a |
| DIO3OS | hsa-miR-520be |
| DIO3OS | hsa-miR-520acd-3p |
| DIO3OS | hsa-miR-1378 |
| DIO3OS | hsa-miR-1420ac |
| DIO3OS | hsa-miR-138 |
| DIO3OS | hsa-miR-138ab |
| DIO3OS | hsa-miR-139-5p |
| DIO3OS | hsa-miR-143 |
| DIO3OS | hsa-miR-1721 |
| DIO3OS | hsa-miR-4770 |
| DIO3OS | hsa-miR-146ac |
| DIO3OS | hsa-miR-146b-5p |
| DIO3OS | hsa-miR-150 |
| DIO3OS | hsa-miR-5127 |
| DIO3OS | hsa-miR-181abcd |
| DIO3OS | hsa-miR-4262 |
| DIO3OS | hsa-miR-let-7 |
| DIO3OS | hsa-miR-98 |
| DIO3OS | hsa-miR-4458 |
| DIO3OS | hsa-miR-4500 |
| DIO3OS | hsa-miR-18ab |
| DIO3OS | hsa-miR-4735-3p |
| DIO3OS | hsa-miR-192 |
| DIO3OS | hsa-miR-215 |
| DIO3OS | hsa-miR-199ab-5p |
| DIO3OS | hsa-miR-19ab |
| DIO3OS | hsa-miR-1ab |
| DIO3OS | hsa-miR-206 |
| DIO3OS | hsa-miR-613 |
| DIO3OS | hsa-miR-204 |
| DIO3OS | hsa-miR-204b |
| DIO3OS | hsa-miR-211 |
| DIO3OS | hsa-miR-214 |
| DIO3OS | hsa-miR-761 |
| DIO3OS | hsa-miR-3619-5p |
| DIO3OS | hsa-miR-217 |
| DIO3OS | hsa-miR-218 |
| DIO3OS | hsa-miR-218a |
| DIO3OS | hsa-miR-219-5p |
| DIO3OS | hsa-miR-508 |
| DIO3OS | hsa-miR-508-3p |
| DIO3OS | hsa-miR-4782-3p |
| DIO3OS | hsa-miR-221 |
| DIO3OS | hsa-miR-222 |
| DIO3OS | hsa-miR-222ab |
| DIO3OS | hsa-miR-1928 |
| DIO3OS | hsa-miR-122 |
| DIO3OS | hsa-miR-122a |
| DIO3OS | hsa-miR-1352 |
| DIO3OS | hsa-miR-24 |
| DIO3OS | hsa-miR-24ab |
| DIO3OS | hsa-miR-24-3p |
| DIO3OS | hsa-miR-27abc |
| DIO3OS | hsa-miR-27a-3p |
| DIO3OS | hsa-miR-124 |
| DIO3OS | hsa-miR-124ab |
| DIO3OS | hsa-miR-506 |
| DIO3OS | hsa-miR-338 |
| DIO3OS | hsa-miR-338-3p |
| DIO3OS | hsa-miR-34ac |
| DIO3OS | hsa-miR-34bc-5p |
| DIO3OS | hsa-miR-449abc |
| DIO3OS | hsa-miR-449c-5p |
| DIO3OS | hsa-miR-383 |
| DIO3OS | hsa-miR-125a-5p |
| DIO3OS | hsa-miR-125b-5p |
| DIO3OS | hsa-miR-351 |
| DIO3OS | hsa-miR-670 |
| DIO3OS | hsa-miR-4319 |
| DIO3OS | hsa-miR-10abc |
| DIO3OS | hsa-miR-10a-5p |
| DIO3OS | hsa-miR-455-5p |
| ARHGAP5-AS1 | hsa-miR-140 |
| ARHGAP5-AS1 | hsa-miR-140-5p |
| ARHGAP5-AS1 | hsa-miR-876-3p |
| ARHGAP5-AS1 | hsa-miR-1244 |
| ARHGAP5-AS1 | hsa-miR-148ab-3p |
| ARHGAP5-AS1 | hsa-miR-152 |
| ARHGAP5-AS1 | hsa-miR-155 |
| ARHGAP5-AS1 | hsa-miR-194 |
| ARHGAP5-AS1 | hsa-miR-199ab-5p |
| ARHGAP5-AS1 | hsa-miR-19ab |
| ARHGAP5-AS1 | hsa-miR-27abc |
| ARHGAP5-AS1 | hsa-miR-27a-3p |
| ARHGAP5-AS1 | hsa-miR-124 |
| ARHGAP5-AS1 | hsa-miR-124ab |
| ARHGAP5-AS1 | hsa-miR-506 |
| ARHGAP5-AS1 | hsa-miR-34ac |
| ARHGAP5-AS1 | hsa-miR-34bc-5p |
| ARHGAP5-AS1 | hsa-miR-449abc |
| ARHGAP5-AS1 | hsa-miR-449c-5p |
| ARHGAP5-AS1 | hsa-miR-125a-5p |
| ARHGAP5-AS1 | hsa-miR-125b-5p |
| ARHGAP5-AS1 | hsa-miR-351 |
| ARHGAP5-AS1 | hsa-miR-670 |
| ARHGAP5-AS1 | hsa-miR-4319 |
| LINC00052 | hsa-miR-145 |
| LINC00052 | hsa-miR-148ab-3p |
| LINC00052 | hsa-miR-152 |
| LINC00052 | hsa-miR-17 |
| LINC00052 | hsa-miR-17-5p |
| LINC00052 | hsa-miR-20ab |
| LINC00052 | hsa-miR-20b-5p |
| LINC00052 | hsa-miR-93 |
| LINC00052 | hsa-miR-106ab |
| LINC00052 | hsa-miR-427 |
| LINC00052 | hsa-miR-518a-3p |
| LINC00052 | hsa-miR-519d |
| LINC00052 | hsa-miR-let-7 |
| LINC00052 | hsa-miR-98 |
| LINC00052 | hsa-miR-4458 |
| LINC00052 | hsa-miR-4500 |
| LINC00052 | hsa-miR-187 |
| LINC00052 | hsa-miR-18ab |
| LINC00052 | hsa-miR-4735-3p |
| LINC00052 | hsa-miR-196abc |
| LINC00052 | hsa-miR-203 |
| LINC00052 | hsa-miR-216b |
| LINC00052 | hsa-miR-216b-5p |
| LINC00052 | hsa-miR-217 |
| LINC00052 | hsa-miR-218 |
| LINC00052 | hsa-miR-218a |
| LINC00052 | hsa-miR-23abc |
| LINC00052 | hsa-miR-23b-3p |
| LINC00052 | hsa-miR-24 |
| LINC00052 | hsa-miR-24ab |
| LINC00052 | hsa-miR-24-3p |
| LINC00052 | hsa-miR-25 |
| LINC00052 | hsa-miR-32 |
| LINC00052 | hsa-miR-92abc |
| LINC00052 | hsa-miR-363 |
| LINC00052 | hsa-miR-363-3p |
| LINC00052 | hsa-miR-367 |
| LINC00052 | hsa-miR-31 |
| LINC00052 | hsa-miR-33ab |
| LINC00052 | hsa-miR-33-5p |
| LINC00052 | hsa-miR-425 |
| LINC00052 | hsa-miR-425-5p |
| LINC00052 | hsa-miR-489 |
| LINC00052 | hsa-miR-10abc |
| LINC00052 | hsa-miR-10a-5p |
| LINC00052 | hsa-miR-455-5p |
| VENTXP1 | hsa-miR-132 |
| VENTXP1 | hsa-miR-212 |
| VENTXP1 | hsa-miR-212-3p |
| VENTXP1 | hsa-miR-93 |
| VENTXP1 | hsa-miR-93a |
| VENTXP1 | hsa-miR-105 |
| VENTXP1 | hsa-miR-106a |
| VENTXP1 | hsa-miR-291a-3p |
| VENTXP1 | hsa-miR-294 |
| VENTXP1 | hsa-miR-295 |
| VENTXP1 | hsa-miR-302abcde |
| VENTXP1 | hsa-miR-372 |
| VENTXP1 | hsa-miR-373 |
| VENTXP1 | hsa-miR-428 |
| VENTXP1 | hsa-miR-519a |
| VENTXP1 | hsa-miR-520be |
| VENTXP1 | hsa-miR-520acd-3p |
| VENTXP1 | hsa-miR-1378 |
| VENTXP1 | hsa-miR-1420ac |
| VENTXP1 | hsa-miR-138 |
| VENTXP1 | hsa-miR-138ab |
| VENTXP1 | hsa-miR-139-5p |
| VENTXP1 | hsa-miR-141 |
| VENTXP1 | hsa-miR-200a |
| VENTXP1 | hsa-miR-144 |
| VENTXP1 | hsa-miR-153 |
| VENTXP1 | hsa-miR-155 |
| VENTXP1 | hsa-miR-let-7 |
| VENTXP1 | hsa-miR-98 |
| VENTXP1 | hsa-miR-4458 |
| VENTXP1 | hsa-miR-4500 |
| VENTXP1 | hsa-miR-204 |
| VENTXP1 | hsa-miR-204b |
| VENTXP1 | hsa-miR-211 |
| VENTXP1 | hsa-miR-205 |
| VENTXP1 | hsa-miR-205ab |
| VENTXP1 | hsa-miR-21 |
| VENTXP1 | hsa-miR-590-5p |
| VENTXP1 | hsa-miR-217 |
| VENTXP1 | hsa-miR-22 |
| VENTXP1 | hsa-miR-22-3p |
| VENTXP1 | hsa-miR-23abc |
| VENTXP1 | hsa-miR-23b-3p |
| VENTXP1 | hsa-miR-25 |
| VENTXP1 | hsa-miR-32 |
| VENTXP1 | hsa-miR-92abc |
| VENTXP1 | hsa-miR-363 |
| VENTXP1 | hsa-miR-363-3p |
| VENTXP1 | hsa-miR-367 |
| VENTXP1 | hsa-miR-27abc |
| VENTXP1 | hsa-miR-27a-3p |
| VENTXP1 | hsa-miR-30abcdef |
| VENTXP1 | hsa-miR-30abe-5p |
| VENTXP1 | hsa-miR-384-5p |
| VENTXP1 | hsa-miR-33a-3p |
| VENTXP1 | hsa-miR-365 |
| VENTXP1 | hsa-miR-365-3p |
| VENTXP1 | hsa-miR-33ab |
| VENTXP1 | hsa-miR-33-5p |
| VENTXP1 | hsa-miR-375 |
| VENTXP1 | hsa-miR-10abc |
| VENTXP1 | hsa-miR-10a-5p |
| VENTXP1 | hsa-miR-128 |
| VENTXP1 | hsa-miR-128ab |
| VENTXP1 | hsa-miR-129-5p |
| VENTXP1 | hsa-miR-129ab-5p |
| VENTXP1 | hsa-miR-490-3p |
| VENTXP1 | hsa-miR-499-5p |
| LINC00261 | hsa-miR-130ac |
| LINC00261 | hsa-miR-301ab |
| LINC00261 | hsa-miR-301b |
| LINC00261 | hsa-miR-301b-3p |
| LINC00261 | hsa-miR-454 |
| LINC00261 | hsa-miR-721 |
| LINC00261 | hsa-miR-4295 |
| LINC00261 | hsa-miR-3666 |
| LINC00261 | hsa-miR-132 |
| LINC00261 | hsa-miR-212 |
| LINC00261 | hsa-miR-212-3p |
| LINC00261 | hsa-miR-135ab |
| LINC00261 | hsa-miR-135a-5p |
| LINC00261 | hsa-miR-139-5p |
| LINC00261 | hsa-miR-140 |
| LINC00261 | hsa-miR-140-5p |
| LINC00261 | hsa-miR-876-3p |
| LINC00261 | hsa-miR-1244 |
| LINC00261 | hsa-miR-143 |
| LINC00261 | hsa-miR-1721 |
| LINC00261 | hsa-miR-4770 |
| LINC00261 | hsa-miR-144 |
| LINC00261 | hsa-miR-145 |
| LINC00261 | hsa-miR-146ac |
| LINC00261 | hsa-miR-146b-5p |
| LINC00261 | hsa-miR-148ab-3p |
| LINC00261 | hsa-miR-152 |
| LINC00261 | hsa-miR-150 |
| LINC00261 | hsa-miR-5127 |
| LINC00261 | hsa-miR-153 |
| LINC00261 | hsa-miR-155 |
| LINC00261 | hsa-miR-182 |
| LINC00261 | hsa-miR-183 |
| LINC00261 | hsa-miR-19ab |
| LINC00261 | hsa-miR-1ab |
| LINC00261 | hsa-miR-206 |
| LINC00261 | hsa-miR-613 |
| LINC00261 | hsa-miR-200bc |
| LINC00261 | hsa-miR-429 |
| LINC00261 | hsa-miR-548a |
| LINC00261 | hsa-miR-204 |
| LINC00261 | hsa-miR-204b |
| LINC00261 | hsa-miR-211 |
| LINC00261 | hsa-miR-214 |
| LINC00261 | hsa-miR-761 |
| LINC00261 | hsa-miR-3619-5p |
| LINC00261 | hsa-miR-216b |
| LINC00261 | hsa-miR-216b-5p |
| LINC00261 | hsa-miR-218 |
| LINC00261 | hsa-miR-218a |
| LINC00261 | hsa-miR-219-5p |
| LINC00261 | hsa-miR-508 |
| LINC00261 | hsa-miR-508-3p |
| LINC00261 | hsa-miR-4782-3p |
| LINC00261 | hsa-miR-223 |
| LINC00261 | hsa-miR-23abc |
| LINC00261 | hsa-miR-23b-3p |
| LINC00261 | hsa-miR-27abc |
| LINC00261 | hsa-miR-27a-3p |
| LINC00261 | hsa-miR-101 |
| LINC00261 | hsa-miR-101ab |
| LINC00261 | hsa-miR-30abcdef |
| LINC00261 | hsa-miR-30abe-5p |
| LINC00261 | hsa-miR-384-5p |
| LINC00261 | hsa-miR-31 |
| LINC00261 | hsa-miR-103a |
| LINC00261 | hsa-miR-107 |
| LINC00261 | hsa-miR-107ab |
| LINC00261 | hsa-miR-124 |
| LINC00261 | hsa-miR-124ab |
| LINC00261 | hsa-miR-506 |
| LINC00261 | hsa-miR-338 |
| LINC00261 | hsa-miR-338-3p |
| LINC00261 | hsa-miR-33ab |
| LINC00261 | hsa-miR-33-5p |
| LINC00261 | hsa-miR-34ac |
| LINC00261 | hsa-miR-34bc-5p |
| LINC00261 | hsa-miR-449abc |
| LINC00261 | hsa-miR-449c-5p |
| LINC00261 | hsa-miR-375 |
| LINC00261 | hsa-miR-125a-5p |
| LINC00261 | hsa-miR-125b-5p |
| LINC00261 | hsa-miR-351 |
| LINC00261 | hsa-miR-670 |
| LINC00261 | hsa-miR-4319 |
| LINC00261 | hsa-miR-10abc |
| LINC00261 | hsa-miR-10a-5p |
| LINC00261 | hsa-miR-128 |
| LINC00261 | hsa-miR-128ab |
| LINC00261 | hsa-miR-129-5p |
| LINC00261 | hsa-miR-129ab-5p |
| LINC00261 | hsa-miR-499-5p |
| FBXL19-AS1 | hsa-miR-9 |
| FBXL19-AS1 | hsa-miR-9ab |
| FBXL19-AS1 | hsa-miR-93 |
| FBXL19-AS1 | hsa-miR-93a |
| FBXL19-AS1 | hsa-miR-105 |
| FBXL19-AS1 | hsa-miR-106a |
| FBXL19-AS1 | hsa-miR-291a-3p |
| FBXL19-AS1 | hsa-miR-294 |
| FBXL19-AS1 | hsa-miR-295 |
| FBXL19-AS1 | hsa-miR-302abcde |
| FBXL19-AS1 | hsa-miR-372 |
| FBXL19-AS1 | hsa-miR-373 |
| FBXL19-AS1 | hsa-miR-428 |
| FBXL19-AS1 | hsa-miR-519a |
| FBXL19-AS1 | hsa-miR-520be |
| FBXL19-AS1 | hsa-miR-520acd-3p |
| FBXL19-AS1 | hsa-miR-1378 |
| FBXL19-AS1 | hsa-miR-1420ac |
| FBXL19-AS1 | hsa-miR-135ab |
| FBXL19-AS1 | hsa-miR-135a-5p |
| FBXL19-AS1 | hsa-miR-138 |
| FBXL19-AS1 | hsa-miR-138ab |
| FBXL19-AS1 | hsa-miR-141 |
| FBXL19-AS1 | hsa-miR-200a |
| FBXL19-AS1 | hsa-miR-142-3p |
| FBXL19-AS1 | hsa-miR-143 |
| FBXL19-AS1 | hsa-miR-1721 |
| FBXL19-AS1 | hsa-miR-4770 |
| FBXL19-AS1 | hsa-miR-148ab-3p |
| FBXL19-AS1 | hsa-miR-152 |
| FBXL19-AS1 | hsa-miR-150 |
| FBXL19-AS1 | hsa-miR-5127 |
| FBXL19-AS1 | hsa-miR-153 |
| FBXL19-AS1 | hsa-miR-17 |
| FBXL19-AS1 | hsa-miR-17-5p |
| FBXL19-AS1 | hsa-miR-20ab |
| FBXL19-AS1 | hsa-miR-20b-5p |
| FBXL19-AS1 | hsa-miR-106ab |
| FBXL19-AS1 | hsa-miR-427 |
| FBXL19-AS1 | hsa-miR-518a-3p |
| FBXL19-AS1 | hsa-miR-519d |
| FBXL19-AS1 | hsa-miR-193 |
| FBXL19-AS1 | hsa-miR-193b |
| FBXL19-AS1 | hsa-miR-193a-3p |
| FBXL19-AS1 | hsa-miR-194 |
| FBXL19-AS1 | hsa-miR-196abc |
| FBXL19-AS1 | hsa-miR-203 |
| FBXL19-AS1 | hsa-miR-214 |
| FBXL19-AS1 | hsa-miR-761 |
| FBXL19-AS1 | hsa-miR-3619-5p |
| FBXL19-AS1 | hsa-miR-216b |
| FBXL19-AS1 | hsa-miR-216b-5p |
| FBXL19-AS1 | hsa-miR-218 |
| FBXL19-AS1 | hsa-miR-218a |
| FBXL19-AS1 | hsa-miR-22 |
| FBXL19-AS1 | hsa-miR-22-3p |
| FBXL19-AS1 | hsa-miR-223 |
| FBXL19-AS1 | hsa-miR-122 |
| FBXL19-AS1 | hsa-miR-122a |
| FBXL19-AS1 | hsa-miR-1352 |
| FBXL19-AS1 | hsa-miR-23abc |
| FBXL19-AS1 | hsa-miR-23b-3p |
| FBXL19-AS1 | hsa-miR-24 |
| FBXL19-AS1 | hsa-miR-24ab |
| FBXL19-AS1 | hsa-miR-24-3p |
| FBXL19-AS1 | hsa-miR-26ab |
| FBXL19-AS1 | hsa-miR-1297 |
| FBXL19-AS1 | hsa-miR-4465 |
| FBXL19-AS1 | hsa-miR-103a |
| FBXL19-AS1 | hsa-miR-107 |
| FBXL19-AS1 | hsa-miR-107ab |
| FBXL19-AS1 | hsa-miR-125a-5p |
| FBXL19-AS1 | hsa-miR-125b-5p |
| FBXL19-AS1 | hsa-miR-351 |
| FBXL19-AS1 | hsa-miR-670 |
| FBXL19-AS1 | hsa-miR-4319 |
| FBXL19-AS1 | hsa-miR-129-5p |
| FBXL19-AS1 | hsa-miR-129ab-5p |
