# Supplementary material for: Development of a prognostic model based on the ceRNA network in Triple-Negative Breast cancer
Source: PeerJ. 2025 Feb 27;13:e19063. doi: 10.7717/peerj.19063 (PMC11874946; doi:10.7717/peerj.19063)
Supplement: Table S2 [file peerj-13-19063-s008.docx]

**TableS2 The Relationships between mRNAs and miRNA**

| **mRNAs** | **miRNAs** |
| --- | --- |
| TGFBR3 | hsa-let-7a-5p |
| COL5A2 | hsa-let-7a-5p |
| RRM2 | hsa-let-7a-5p |
| COL3A1 | hsa-let-7a-5p |
| TTLL4 | hsa-let-7a-5p |
| DUSP1 | hsa-let-7a-5p |
| ADRB2 | hsa-let-7a-5p |
| AP1S1 | hsa-let-7a-5p |
| TGFBR3 | hsa-let-7b-5p |
| COL5A2 | hsa-let-7b-5p |
| RRM2 | hsa-let-7b-5p |
| COL3A1 | hsa-let-7b-5p |
| TTLL4 | hsa-let-7b-5p |
| DUSP1 | hsa-let-7b-5p |
| ADRB2 | hsa-let-7b-5p |
| AP1S1 | hsa-let-7b-5p |
| TGFBR3 | hsa-let-7c-5p |
| COL5A2 | hsa-let-7c-5p |
| RRM2 | hsa-let-7c-5p |
| COL3A1 | hsa-let-7c-5p |
| TTLL4 | hsa-let-7c-5p |
| DUSP1 | hsa-let-7c-5p |
| ADRB2 | hsa-let-7c-5p |
| AP1S1 | hsa-let-7c-5p |
| TGFBR3 | hsa-let-7d-5p |
| COL5A2 | hsa-let-7d-5p |
| RRM2 | hsa-let-7d-5p |
| COL3A1 | hsa-let-7d-5p |
| TTLL4 | hsa-let-7d-5p |
| DUSP1 | hsa-let-7d-5p |
| ADRB2 | hsa-let-7d-5p |
| AP1S1 | hsa-let-7d-5p |
| TGFBR3 | hsa-let-7e-5p |
| COL5A2 | hsa-let-7e-5p |
| RRM2 | hsa-let-7e-5p |
| COL3A1 | hsa-let-7e-5p |
| TTLL4 | hsa-let-7e-5p |
| DUSP1 | hsa-let-7e-5p |
| ADRB2 | hsa-let-7e-5p |
| AP1S1 | hsa-let-7e-5p |
| TGFBR3 | hsa-let-7f-5p |
| COL5A2 | hsa-let-7f-5p |
| RRM2 | hsa-let-7f-5p |
| COL3A1 | hsa-let-7f-5p |
| TTLL4 | hsa-let-7f-5p |
| DUSP1 | hsa-let-7f-5p |
| ADRB2 | hsa-let-7f-5p |
| AP1S1 | hsa-let-7f-5p |
| TGFBR3 | hsa-let-7g-5p |
| COL5A2 | hsa-let-7g-5p |
| RRM2 | hsa-let-7g-5p |
| COL3A1 | hsa-let-7g-5p |
| TTLL4 | hsa-let-7g-5p |
| DUSP1 | hsa-let-7g-5p |
| ADRB2 | hsa-let-7g-5p |
| AP1S1 | hsa-let-7g-5p |
| TGFBR3 | hsa-let-7i-5p |
| COL5A2 | hsa-let-7i-5p |
| RRM2 | hsa-let-7i-5p |
| COL3A1 | hsa-let-7i-5p |
| TTLL4 | hsa-let-7i-5p |
| DUSP1 | hsa-let-7i-5p |
| ADRB2 | hsa-let-7i-5p |
| AP1S1 | hsa-let-7i-5p |
| ANP32E | hsa-miR-1-3p |
| ADAR | hsa-miR-1-3p |
| RRBP1 | hsa-miR-1-3p |
| STC2 | hsa-miR-1-3p |
| KDELR2 | hsa-miR-1-3p |
| AP1S1 | hsa-miR-1-3p |
| STMN1 | hsa-miR-101-3p |
| TGFBR3 | hsa-miR-101-3p |
| DDIT4 | hsa-miR-101-3p |
| FOXO1 | hsa-miR-101-3p |
| KCTD12 | hsa-miR-101-3p |
| FOS | hsa-miR-101-3p |
| AXIN2 | hsa-miR-101-3p |
| DNMT3A | hsa-miR-101-3p |
| FAT4 | hsa-miR-101-3p |
| DUSP1 | hsa-miR-101-3p |
| H2AFV | hsa-miR-101-3p |
| TGFBR3 | hsa-miR-103a-3p |
| AXIN2 | hsa-miR-103a-3p |
| FAT4 | hsa-miR-103a-3p |
| CALU | hsa-miR-103a-3p |
| CAMK2N1 | hsa-miR-106a-5p |
| F3 | hsa-miR-106a-5p |
| ADAR | hsa-miR-106a-5p |
| S1PR1 | hsa-miR-106a-5p |
| MASTL | hsa-miR-106a-5p |
| ATL3 | hsa-miR-106a-5p |
| TMEM123 | hsa-miR-106a-5p |
| TMEM25 | hsa-miR-106a-5p |
| KPNA2 | hsa-miR-106a-5p |
| RND3 | hsa-miR-106a-5p |
| SLC40A1 | hsa-miR-106a-5p |
| RRM2 | hsa-miR-106a-5p |
| LRIG1 | hsa-miR-106a-5p |
| TIPARP | hsa-miR-106a-5p |
| CCNG2 | hsa-miR-106a-5p |
| SOBP | hsa-miR-106a-5p |
| ETV1 | hsa-miR-106a-5p |
| ARL4A | hsa-miR-106a-5p |
| HMBOX1 | hsa-miR-106a-5p |
| RNF128 | hsa-miR-106a-5p |
| CAMK2N1 | hsa-miR-106b-5p |
| F3 | hsa-miR-106b-5p |
| ADAR | hsa-miR-106b-5p |
| S1PR1 | hsa-miR-106b-5p |
| MASTL | hsa-miR-106b-5p |
| ATL3 | hsa-miR-106b-5p |
| TMEM123 | hsa-miR-106b-5p |
| TMEM25 | hsa-miR-106b-5p |
| KPNA2 | hsa-miR-106b-5p |
| RND3 | hsa-miR-106b-5p |
| SLC40A1 | hsa-miR-106b-5p |
| RRM2 | hsa-miR-106b-5p |
| LRIG1 | hsa-miR-106b-5p |
| TIPARP | hsa-miR-106b-5p |
| CCNG2 | hsa-miR-106b-5p |
| SOBP | hsa-miR-106b-5p |
| ETV1 | hsa-miR-106b-5p |
| ARL4A | hsa-miR-106b-5p |
| HMBOX1 | hsa-miR-106b-5p |
| RNF128 | hsa-miR-106b-5p |
| TGFBR3 | hsa-miR-107 |
| AXIN2 | hsa-miR-107 |
| FAT4 | hsa-miR-107 |
| CALU | hsa-miR-107 |
| RAP2A | hsa-miR-10a-5p |
| SDC1 | hsa-miR-10a-5p |
| SOBP | hsa-miR-10a-5p |
| RAP2A | hsa-miR-10b-5p |
| SDC1 | hsa-miR-10b-5p |
| SOBP | hsa-miR-10b-5p |
| ATL3 | hsa-miR-124-3p |
| DUSP6 | hsa-miR-124-3p |
| SPRY2 | hsa-miR-124-3p |
| RRBP1 | hsa-miR-124-3p |
| KDELR2 | hsa-miR-124-3p |
| GLI3 | hsa-miR-124-3p |
| GNAI1 | hsa-miR-124-3p |
| ARPC1B | hsa-miR-124-3p |
| CAV1 | hsa-miR-124-3p |
| CALU | hsa-miR-124-3p |
| TMEM123 | hsa-miR-125a-5p |
| FAT4 | hsa-miR-125a-5p |
| BAK1 | hsa-miR-125a-5p |
| TMEM123 | hsa-miR-125b-5p |
| FAT4 | hsa-miR-125b-5p |
| BAK1 | hsa-miR-125b-5p |
| CAMK2N1 | hsa-miR-1271-5p |
| DEPDC1 | hsa-miR-1271-5p |
| RGS2 | hsa-miR-1271-5p |
| FOXO1 | hsa-miR-1271-5p |
| ABAT | hsa-miR-1271-5p |
| RND3 | hsa-miR-1271-5p |
| PDK1 | hsa-miR-1271-5p |
| LRIG1 | hsa-miR-1271-5p |
| AMOTL2 | hsa-miR-1271-5p |
| CCNG2 | hsa-miR-1271-5p |
| EZR | hsa-miR-1271-5p |
| F3 | hsa-miR-128-3p |
| CCT3 | hsa-miR-128-3p |
| SPRY2 | hsa-miR-128-3p |
| PRKD1 | hsa-miR-128-3p |
| CA12 | hsa-miR-128-3p |
| RND3 | hsa-miR-128-3p |
| PDK1 | hsa-miR-128-3p |
| COL3A1 | hsa-miR-128-3p |
| CAMK2N1 | hsa-miR-129-5p |
| HDGF | hsa-miR-129-5p |
| KCNK1 | hsa-miR-129-5p |
| DNMT3A | hsa-miR-129-5p |
| AMOTL2 | hsa-miR-129-5p |
| ETV1 | hsa-miR-129-5p |
| STMN1 | hsa-miR-1297 |
| DEPDC1 | hsa-miR-1297 |
| FLVCR1 | hsa-miR-1297 |
| KCNK1 | hsa-miR-1297 |
| IGF1 | hsa-miR-1297 |
| KPNA2 | hsa-miR-1297 |
| INHBB | hsa-miR-1297 |
| C20orf24 | hsa-miR-1297 |
| NAP1L5 | hsa-miR-1297 |
| PTPN13 | hsa-miR-1297 |
| DCDC2 | hsa-miR-1297 |
| LOXL2 | hsa-miR-1297 |
| CSTF2 | hsa-miR-1297 |
| LRP8 | hsa-miR-130a-3p |
| DEPDC1 | hsa-miR-130a-3p |
| F3 | hsa-miR-130a-3p |
| S1PR1 | hsa-miR-130a-3p |
| CEP55 | hsa-miR-130a-3p |
| IGF1 | hsa-miR-130a-3p |
| NPTX1 | hsa-miR-130a-3p |
| INHBB | hsa-miR-130a-3p |
| LRIG1 | hsa-miR-130a-3p |
| KIT | hsa-miR-130a-3p |
| LRP8 | hsa-miR-130b-3p |
| DEPDC1 | hsa-miR-130b-3p |
| F3 | hsa-miR-130b-3p |
| S1PR1 | hsa-miR-130b-3p |
| CEP55 | hsa-miR-130b-3p |
| IGF1 | hsa-miR-130b-3p |
| NPTX1 | hsa-miR-130b-3p |
| INHBB | hsa-miR-130b-3p |
| LRIG1 | hsa-miR-130b-3p |
| KIT | hsa-miR-130b-3p |
| FOXO1 | hsa-miR-132-3p |
| DNMT3A | hsa-miR-132-3p |
| RND3 | hsa-miR-132-3p |
| H2AFZ | hsa-miR-132-3p |
| SPRY1 | hsa-miR-132-3p |
| ETV1 | hsa-miR-132-3p |
| CALU | hsa-miR-132-3p |
| FLVCR1 | hsa-miR-133a-3p |
| DUSP1 | hsa-miR-133a-3p |
| FLVCR1 | hsa-miR-133b |
| DUSP1 | hsa-miR-133b |
| ANP32E | hsa-miR-135a-5p |
| ARHGAP11A | hsa-miR-135a-5p |
| ABAT | hsa-miR-135a-5p |
| NPTX1 | hsa-miR-135a-5p |
| CCNG2 | hsa-miR-135a-5p |
| ANP32E | hsa-miR-135b-5p |
| ARHGAP11A | hsa-miR-135b-5p |
| ABAT | hsa-miR-135b-5p |
| NPTX1 | hsa-miR-135b-5p |
| CCNG2 | hsa-miR-135b-5p |
| MPZL1 | hsa-miR-136-5p |
| CXCL12 | hsa-miR-137 |
| CCNG2 | hsa-miR-137 |
| STC2 | hsa-miR-137 |
| BAIAP2L1 | hsa-miR-137 |
| CAMK2N1 | hsa-miR-138-5p |
| PDK1 | hsa-miR-138-5p |
| TIPARP | hsa-miR-138-5p |
| KDELR2 | hsa-miR-138-5p |
| HDGF | hsa-miR-139-5p |
| DDIT4 | hsa-miR-139-5p |
| FOS | hsa-miR-139-5p |
| NPTX1 | hsa-miR-139-5p |
| H2AFV | hsa-miR-139-5p |
| CAMK2N1 | hsa-miR-140-5p |
| TMEM123 | hsa-miR-140-5p |
| STC2 | hsa-miR-140-5p |
| CALU | hsa-miR-140-5p |
| ANP32E | hsa-miR-141-3p |
| CXCL12 | hsa-miR-141-3p |
| KCTD12 | hsa-miR-141-3p |
| H2AFZ | hsa-miR-141-3p |
| HMGCS1 | hsa-miR-141-3p |
| DUSP1 | hsa-miR-141-3p |
| HNRNPAB | hsa-miR-141-3p |
| SOBP | hsa-miR-141-3p |
| ARL4A | hsa-miR-141-3p |
| CALU | hsa-miR-141-3p |
| FLVCR1 | hsa-miR-142-3p |
| CNIH4 | hsa-miR-142-3p |
| TIPARP | hsa-miR-142-3p |
| KDELR2 | hsa-miR-142-3p |
| COL5A2 | hsa-miR-143-3p |
| MYBL2 | hsa-miR-143-3p |
| SOBP | hsa-miR-143-3p |
| GLI3 | hsa-miR-143-3p |
| STMN1 | hsa-miR-144-3p |
| KIF14 | hsa-miR-144-3p |
| CXCL12 | hsa-miR-144-3p |
| CEP55 | hsa-miR-144-3p |
| BHLHE41 | hsa-miR-144-3p |
| FOXO1 | hsa-miR-144-3p |
| FOS | hsa-miR-144-3p |
| IDH2 | hsa-miR-144-3p |
| TOP2A | hsa-miR-144-3p |
| AXIN2 | hsa-miR-144-3p |
| KPNA2 | hsa-miR-144-3p |
| COL5A2 | hsa-miR-144-3p |
| ANTXR2 | hsa-miR-144-3p |
| CCNG2 | hsa-miR-144-3p |
| FAT4 | hsa-miR-144-3p |
| DUSP1 | hsa-miR-144-3p |
| HNRNPAB | hsa-miR-144-3p |
| SOBP | hsa-miR-144-3p |
| H2AFV | hsa-miR-144-3p |
| MPZL1 | hsa-miR-145-5p |
| FOXO1 | hsa-miR-145-5p |
| CIRBP | hsa-miR-145-5p |
| INHBB | hsa-miR-145-5p |
| PGK1 | hsa-miR-146a-5p |
| PGK1 | hsa-miR-146b-5p |
| F3 | hsa-miR-148a-3p |
| CEP55 | hsa-miR-148a-3p |
| NPTX1 | hsa-miR-148a-3p |
| INHBB | hsa-miR-148a-3p |
| KIT | hsa-miR-148a-3p |
| DUSP1 | hsa-miR-148a-3p |
| F3 | hsa-miR-148b-3p |
| CEP55 | hsa-miR-148b-3p |
| NPTX1 | hsa-miR-148b-3p |
| INHBB | hsa-miR-148b-3p |
| KIT | hsa-miR-148b-3p |
| DUSP1 | hsa-miR-148b-3p |
| CCT3 | hsa-miR-149-5p |
| CNIH4 | hsa-miR-149-5p |
| SHMT2 | hsa-miR-149-5p |
| FOS | hsa-miR-149-5p |
| AMOTL2 | hsa-miR-149-5p |
| CALU | hsa-miR-150-5p |
| F3 | hsa-miR-152-3p |
| CEP55 | hsa-miR-152-3p |
| NPTX1 | hsa-miR-152-3p |
| INHBB | hsa-miR-152-3p |
| KIT | hsa-miR-152-3p |
| DUSP1 | hsa-miR-152-3p |
| DDIT4 | hsa-miR-153-3p |
| TMEM25 | hsa-miR-153-3p |
| INHBB | hsa-miR-153-3p |
| IRX2 | hsa-miR-153-3p |
| ARL4A | hsa-miR-153-3p |
| S1PR1 | hsa-miR-155-5p |
| FOS | hsa-miR-155-5p |
| ANTXR2 | hsa-miR-155-5p |
| HMGCS1 | hsa-miR-155-5p |
| TGFBR3 | hsa-miR-15a-5p |
| HDGF | hsa-miR-15a-5p |
| C1orf21 | hsa-miR-15a-5p |
| CEP55 | hsa-miR-15a-5p |
| CHEK1 | hsa-miR-15a-5p |
| FGF7 | hsa-miR-15a-5p |
| TMEM100 | hsa-miR-15a-5p |
| AXIN2 | hsa-miR-15a-5p |
| LRIG1 | hsa-miR-15a-5p |
| FAT4 | hsa-miR-15a-5p |
| ADRB2 | hsa-miR-15a-5p |
| SH3BGRL2 | hsa-miR-15a-5p |
| SOBP | hsa-miR-15a-5p |
| ANLN | hsa-miR-15a-5p |
| CALU | hsa-miR-15a-5p |
| CA8 | hsa-miR-15a-5p |
| HMBOX1 | hsa-miR-15a-5p |
| TGFBR3 | hsa-miR-15b-5p |
| HDGF | hsa-miR-15b-5p |
| C1orf21 | hsa-miR-15b-5p |
| CEP55 | hsa-miR-15b-5p |
| CHEK1 | hsa-miR-15b-5p |
| FGF7 | hsa-miR-15b-5p |
| TMEM100 | hsa-miR-15b-5p |
| AXIN2 | hsa-miR-15b-5p |
| LRIG1 | hsa-miR-15b-5p |
| FAT4 | hsa-miR-15b-5p |
| ADRB2 | hsa-miR-15b-5p |
| SH3BGRL2 | hsa-miR-15b-5p |
| SOBP | hsa-miR-15b-5p |
| ANLN | hsa-miR-15b-5p |
| CALU | hsa-miR-15b-5p |
| CA8 | hsa-miR-15b-5p |
| HMBOX1 | hsa-miR-15b-5p |
| TGFBR3 | hsa-miR-16-5p |
| HDGF | hsa-miR-16-5p |
| C1orf21 | hsa-miR-16-5p |
| CEP55 | hsa-miR-16-5p |
| CHEK1 | hsa-miR-16-5p |
| FGF7 | hsa-miR-16-5p |
| TMEM100 | hsa-miR-16-5p |
| AXIN2 | hsa-miR-16-5p |
| LRIG1 | hsa-miR-16-5p |
| FAT4 | hsa-miR-16-5p |
| ADRB2 | hsa-miR-16-5p |
| SH3BGRL2 | hsa-miR-16-5p |
| SOBP | hsa-miR-16-5p |
| ANLN | hsa-miR-16-5p |
| CALU | hsa-miR-16-5p |
| CA8 | hsa-miR-16-5p |
| HMBOX1 | hsa-miR-16-5p |
| CAMK2N1 | hsa-miR-17-5p |
| F3 | hsa-miR-17-5p |
| ADAR | hsa-miR-17-5p |
| S1PR1 | hsa-miR-17-5p |
| MASTL | hsa-miR-17-5p |
| ATL3 | hsa-miR-17-5p |
| TMEM123 | hsa-miR-17-5p |
| TMEM25 | hsa-miR-17-5p |
| KPNA2 | hsa-miR-17-5p |
| RND3 | hsa-miR-17-5p |
| SLC40A1 | hsa-miR-17-5p |
| RRM2 | hsa-miR-17-5p |
| LRIG1 | hsa-miR-17-5p |
| TIPARP | hsa-miR-17-5p |
| CCNG2 | hsa-miR-17-5p |
| SOBP | hsa-miR-17-5p |
| ETV1 | hsa-miR-17-5p |
| ARL4A | hsa-miR-17-5p |
| HMBOX1 | hsa-miR-17-5p |
| RNF128 | hsa-miR-17-5p |
| NMT2 | hsa-miR-181a-5p |
| DDIT4 | hsa-miR-181a-5p |
| DUSP6 | hsa-miR-181a-5p |
| FOS | hsa-miR-181a-5p |
| GNB4 | hsa-miR-181a-5p |
| CCNB1 | hsa-miR-181a-5p |
| NMT2 | hsa-miR-181b-5p |
| DDIT4 | hsa-miR-181b-5p |
| DUSP6 | hsa-miR-181b-5p |
| FOS | hsa-miR-181b-5p |
| GNB4 | hsa-miR-181b-5p |
| CCNB1 | hsa-miR-181b-5p |
| NMT2 | hsa-miR-181c-5p |
| DDIT4 | hsa-miR-181c-5p |
| DUSP6 | hsa-miR-181c-5p |
| FOS | hsa-miR-181c-5p |
| GNB4 | hsa-miR-181c-5p |
| CCNB1 | hsa-miR-181c-5p |
| NMT2 | hsa-miR-181d-5p |
| DDIT4 | hsa-miR-181d-5p |
| DUSP6 | hsa-miR-181d-5p |
| FOS | hsa-miR-181d-5p |
| GNB4 | hsa-miR-181d-5p |
| CCNB1 | hsa-miR-181d-5p |
| CAMK2N1 | hsa-miR-182-5p |
| HMCN1 | hsa-miR-182-5p |
| RND3 | hsa-miR-182-5p |
| AMOTL2 | hsa-miR-182-5p |
| DUSP1 | hsa-miR-182-5p |
| ANLN | hsa-miR-182-5p |
| DDIT4 | hsa-miR-183-5p |
| FOXO1 | hsa-miR-183-5p |
| IDH2 | hsa-miR-183-5p |
| CCNB1 | hsa-miR-183-5p |
| EZR | hsa-miR-183-5p |
| SH3BGRL2 | hsa-miR-183-5p |
| IRX2 | hsa-miR-186-5p |
| CENPK | hsa-miR-186-5p |
| KDELR2 | hsa-miR-186-5p |
| GNAI1 | hsa-miR-186-5p |
| CAMK2N1 | hsa-miR-18a-5p |
| F3 | hsa-miR-18a-5p |
| ADD3 | hsa-miR-18a-5p |
| KIT | hsa-miR-18a-5p |
| HMGCS1 | hsa-miR-18a-5p |
| HMBOX1 | hsa-miR-18a-5p |
| CAMK2N1 | hsa-miR-18b-5p |
| F3 | hsa-miR-18b-5p |
| ADD3 | hsa-miR-18b-5p |
| KIT | hsa-miR-18b-5p |
| HMGCS1 | hsa-miR-18b-5p |
| HMBOX1 | hsa-miR-18b-5p |
| CAT | hsa-miR-190a-5p |
| CAT | hsa-miR-190b |
| STMN1 | hsa-miR-193a-3p |
| TGFBR3 | hsa-miR-193a-3p |
| STMN1 | hsa-miR-193b-3p |
| TGFBR3 | hsa-miR-193b-3p |
| STMN1 | hsa-miR-194-5p |
| DUSP1 | hsa-miR-194-5p |
| TGFBR3 | hsa-miR-195-5p |
| HDGF | hsa-miR-195-5p |
| C1orf21 | hsa-miR-195-5p |
| CEP55 | hsa-miR-195-5p |
| CHEK1 | hsa-miR-195-5p |
| FGF7 | hsa-miR-195-5p |
| TMEM100 | hsa-miR-195-5p |
| AXIN2 | hsa-miR-195-5p |
| LRIG1 | hsa-miR-195-5p |
| FAT4 | hsa-miR-195-5p |
| ADRB2 | hsa-miR-195-5p |
| SH3BGRL2 | hsa-miR-195-5p |
| SOBP | hsa-miR-195-5p |
| ANLN | hsa-miR-195-5p |
| CALU | hsa-miR-195-5p |
| CA8 | hsa-miR-195-5p |
| HMBOX1 | hsa-miR-195-5p |
| TGFBR3 | hsa-miR-196a-5p |
| COL3A1 | hsa-miR-196a-5p |
| DCDC2 | hsa-miR-196a-5p |
| TGFBR3 | hsa-miR-196b-5p |
| COL3A1 | hsa-miR-196b-5p |
| DCDC2 | hsa-miR-196b-5p |
| HMCN1 | hsa-miR-199a-5p |
| ADD3 | hsa-miR-199a-5p |
| CAV1 | hsa-miR-199a-5p |
| HMCN1 | hsa-miR-199b-5p |
| ADD3 | hsa-miR-199b-5p |
| CAV1 | hsa-miR-199b-5p |
| F3 | hsa-miR-19a-3p |
| S1PR1 | hsa-miR-19a-3p |
| CEP55 | hsa-miR-19a-3p |
| ADD3 | hsa-miR-19a-3p |
| ACADSB | hsa-miR-19a-3p |
| TMEM25 | hsa-miR-19a-3p |
| ARHGAP11A | hsa-miR-19a-3p |
| SDC1 | hsa-miR-19a-3p |
| INHBB | hsa-miR-19a-3p |
| LRIG1 | hsa-miR-19a-3p |
| TIPARP | hsa-miR-19a-3p |
| KIT | hsa-miR-19a-3p |
| HMGCS1 | hsa-miR-19a-3p |
| F3 | hsa-miR-19b-3p |
| S1PR1 | hsa-miR-19b-3p |
| CEP55 | hsa-miR-19b-3p |
| ADD3 | hsa-miR-19b-3p |
| ACADSB | hsa-miR-19b-3p |
| TMEM25 | hsa-miR-19b-3p |
| ARHGAP11A | hsa-miR-19b-3p |
| SDC1 | hsa-miR-19b-3p |
| INHBB | hsa-miR-19b-3p |
| LRIG1 | hsa-miR-19b-3p |
| TIPARP | hsa-miR-19b-3p |
| KIT | hsa-miR-19b-3p |
| HMGCS1 | hsa-miR-19b-3p |
| ANP32E | hsa-miR-200a-3p |
| CXCL12 | hsa-miR-200a-3p |
| KCTD12 | hsa-miR-200a-3p |
| H2AFZ | hsa-miR-200a-3p |
| HMGCS1 | hsa-miR-200a-3p |
| DUSP1 | hsa-miR-200a-3p |
| HNRNPAB | hsa-miR-200a-3p |
| SOBP | hsa-miR-200a-3p |
| ARL4A | hsa-miR-200a-3p |
| CALU | hsa-miR-200a-3p |
| DDIT4 | hsa-miR-200b-3p |
| ADD3 | hsa-miR-200b-3p |
| ABAT | hsa-miR-200b-3p |
| NPTX1 | hsa-miR-200b-3p |
| RND3 | hsa-miR-200b-3p |
| LRIG1 | hsa-miR-200b-3p |
| AMOTL2 | hsa-miR-200b-3p |
| PTPN13 | hsa-miR-200b-3p |
| DUSP1 | hsa-miR-200b-3p |
| GLI3 | hsa-miR-200b-3p |
| ANLN | hsa-miR-200b-3p |
| CALU | hsa-miR-200b-3p |
| DDIT4 | hsa-miR-200c-3p |
| ADD3 | hsa-miR-200c-3p |
| ABAT | hsa-miR-200c-3p |
| NPTX1 | hsa-miR-200c-3p |
| RND3 | hsa-miR-200c-3p |
| LRIG1 | hsa-miR-200c-3p |
| AMOTL2 | hsa-miR-200c-3p |
| PTPN13 | hsa-miR-200c-3p |
| DUSP1 | hsa-miR-200c-3p |
| GLI3 | hsa-miR-200c-3p |
| ANLN | hsa-miR-200c-3p |
| CALU | hsa-miR-200c-3p |
| RRM2 | hsa-miR-204-5p |
| AP1S1 | hsa-miR-204-5p |
| EZR | hsa-miR-205-5p |
| CALU | hsa-miR-205-5p |
| LYN | hsa-miR-205-5p |
| ANP32E | hsa-miR-206 |
| ADAR | hsa-miR-206 |
| RRBP1 | hsa-miR-206 |
| STC2 | hsa-miR-206 |
| KDELR2 | hsa-miR-206 |
| AP1S1 | hsa-miR-206 |
| H2AFZ | hsa-miR-208a-3p |
| H2AFZ | hsa-miR-208b-3p |
| CAMK2N1 | hsa-miR-20a-5p |
| F3 | hsa-miR-20a-5p |
| ADAR | hsa-miR-20a-5p |
| S1PR1 | hsa-miR-20a-5p |
| MASTL | hsa-miR-20a-5p |
| ATL3 | hsa-miR-20a-5p |
| TMEM123 | hsa-miR-20a-5p |
| TMEM25 | hsa-miR-20a-5p |
| KPNA2 | hsa-miR-20a-5p |
| RND3 | hsa-miR-20a-5p |
| SLC40A1 | hsa-miR-20a-5p |
| RRM2 | hsa-miR-20a-5p |
| LRIG1 | hsa-miR-20a-5p |
| TIPARP | hsa-miR-20a-5p |
| CCNG2 | hsa-miR-20a-5p |
| SOBP | hsa-miR-20a-5p |
| ETV1 | hsa-miR-20a-5p |
| ARL4A | hsa-miR-20a-5p |
| HMBOX1 | hsa-miR-20a-5p |
| RNF128 | hsa-miR-20a-5p |
| CAMK2N1 | hsa-miR-20b-5p |
| F3 | hsa-miR-20b-5p |
| ADAR | hsa-miR-20b-5p |
| S1PR1 | hsa-miR-20b-5p |
| MASTL | hsa-miR-20b-5p |
| ATL3 | hsa-miR-20b-5p |
| TMEM123 | hsa-miR-20b-5p |
| TMEM25 | hsa-miR-20b-5p |
| KPNA2 | hsa-miR-20b-5p |
| RND3 | hsa-miR-20b-5p |
| SLC40A1 | hsa-miR-20b-5p |
| RRM2 | hsa-miR-20b-5p |
| LRIG1 | hsa-miR-20b-5p |
| TIPARP | hsa-miR-20b-5p |
| CCNG2 | hsa-miR-20b-5p |
| SOBP | hsa-miR-20b-5p |
| ETV1 | hsa-miR-20b-5p |
| ARL4A | hsa-miR-20b-5p |
| HMBOX1 | hsa-miR-20b-5p |
| RNF128 | hsa-miR-20b-5p |
| SPRY2 | hsa-miR-21-5p |
| RRM2 | hsa-miR-211-5p |
| AP1S1 | hsa-miR-211-5p |
| FOXO1 | hsa-miR-212-3p |
| DNMT3A | hsa-miR-212-3p |
| RND3 | hsa-miR-212-3p |
| H2AFZ | hsa-miR-212-3p |
| SPRY1 | hsa-miR-212-3p |
| ETV1 | hsa-miR-212-3p |
| CALU | hsa-miR-212-3p |
| DEPDC1 | hsa-miR-216a-5p |
| ANLN | hsa-miR-217 |
| TMEM123 | hsa-miR-218-5p |
| TMEM25 | hsa-miR-218-5p |
| NAV3 | hsa-miR-218-5p |
| LRIG1 | hsa-miR-218-5p |
| KLHL13 | hsa-miR-218-5p |
| CAMK2N1 | hsa-miR-22-3p |
| RGS2 | hsa-miR-22-3p |
| DDIT4 | hsa-miR-22-3p |
| MTHFD2 | hsa-miR-22-3p |
| AP1S1 | hsa-miR-22-3p |
| STMN1 | hsa-miR-221-3p |
| CXCL12 | hsa-miR-221-3p |
| FOS | hsa-miR-221-3p |
| KPNA2 | hsa-miR-221-3p |
| NAP1L5 | hsa-miR-221-3p |
| HMBOX1 | hsa-miR-221-3p |
| STMN1 | hsa-miR-222-3p |
| CXCL12 | hsa-miR-222-3p |
| FOS | hsa-miR-222-3p |
| KPNA2 | hsa-miR-222-3p |
| NAP1L5 | hsa-miR-222-3p |
| HMBOX1 | hsa-miR-222-3p |
| TGFBR3 | hsa-miR-223-3p |
| F3 | hsa-miR-223-3p |
| CCT3 | hsa-miR-223-3p |
| FOXO1 | hsa-miR-223-3p |
| NR4A1 | hsa-miR-224-5p |
| KCTD12 | hsa-miR-224-5p |
| CSTF2 | hsa-miR-224-5p |
| CAMK2N1 | hsa-miR-23a-3p |
| TGFBR3 | hsa-miR-23a-3p |
| CXCL12 | hsa-miR-23a-3p |
| SPRY2 | hsa-miR-23a-3p |
| LRIG1 | hsa-miR-23a-3p |
| NAP1L5 | hsa-miR-23a-3p |
| PDGFA | hsa-miR-23a-3p |
| AP1S1 | hsa-miR-23a-3p |
| CAMK2N1 | hsa-miR-23b-3p |
| TGFBR3 | hsa-miR-23b-3p |
| CXCL12 | hsa-miR-23b-3p |
| SPRY2 | hsa-miR-23b-3p |
| LRIG1 | hsa-miR-23b-3p |
| NAP1L5 | hsa-miR-23b-3p |
| PDGFA | hsa-miR-23b-3p |
| AP1S1 | hsa-miR-23b-3p |
| CCT3 | hsa-miR-24-3p |
| ELF3 | hsa-miR-24-3p |
| AMOTL2 | hsa-miR-24-3p |
| STC2 | hsa-miR-24-3p |
| ANP32E | hsa-miR-25-3p |
| DDIT4 | hsa-miR-25-3p |
| DKK3 | hsa-miR-25-3p |
| DUSP6 | hsa-miR-25-3p |
| MTHFD2 | hsa-miR-25-3p |
| RRBP1 | hsa-miR-25-3p |
| AURKA | hsa-miR-25-3p |
| BAK1 | hsa-miR-25-3p |
| SOBP | hsa-miR-25-3p |
| MMP16 | hsa-miR-25-3p |
| STMN1 | hsa-miR-26a-5p |
| DEPDC1 | hsa-miR-26a-5p |
| FLVCR1 | hsa-miR-26a-5p |
| KCNK1 | hsa-miR-26a-5p |
| IGF1 | hsa-miR-26a-5p |
| KPNA2 | hsa-miR-26a-5p |
| INHBB | hsa-miR-26a-5p |
| C20orf24 | hsa-miR-26a-5p |
| NAP1L5 | hsa-miR-26a-5p |
| PTPN13 | hsa-miR-26a-5p |
| DCDC2 | hsa-miR-26a-5p |
| LOXL2 | hsa-miR-26a-5p |
| CSTF2 | hsa-miR-26a-5p |
| STMN1 | hsa-miR-26b-5p |
| DEPDC1 | hsa-miR-26b-5p |
| FLVCR1 | hsa-miR-26b-5p |
| KCNK1 | hsa-miR-26b-5p |
| IGF1 | hsa-miR-26b-5p |
| KPNA2 | hsa-miR-26b-5p |
| INHBB | hsa-miR-26b-5p |
| C20orf24 | hsa-miR-26b-5p |
| NAP1L5 | hsa-miR-26b-5p |
| PTPN13 | hsa-miR-26b-5p |
| DCDC2 | hsa-miR-26b-5p |
| LOXL2 | hsa-miR-26b-5p |
| CSTF2 | hsa-miR-26b-5p |
| TGFBR3 | hsa-miR-27a-3p |
| F3 | hsa-miR-27a-3p |
| ADD3 | hsa-miR-27a-3p |
| TMEM123 | hsa-miR-27a-3p |
| FOXO1 | hsa-miR-27a-3p |
| SPRY2 | hsa-miR-27a-3p |
| RND3 | hsa-miR-27a-3p |
| TFPI | hsa-miR-27a-3p |
| PDK1 | hsa-miR-27a-3p |
| AMOTL2 | hsa-miR-27a-3p |
| H2AFZ | hsa-miR-27a-3p |
| HMGCS1 | hsa-miR-27a-3p |
| SH3BGRL2 | hsa-miR-27a-3p |
| TGFBR3 | hsa-miR-27b-3p |
| F3 | hsa-miR-27b-3p |
| ADD3 | hsa-miR-27b-3p |
| TMEM123 | hsa-miR-27b-3p |
| FOXO1 | hsa-miR-27b-3p |
| SPRY2 | hsa-miR-27b-3p |
| RND3 | hsa-miR-27b-3p |
| TFPI | hsa-miR-27b-3p |
| PDK1 | hsa-miR-27b-3p |
| AMOTL2 | hsa-miR-27b-3p |
| H2AFZ | hsa-miR-27b-3p |
| HMGCS1 | hsa-miR-27b-3p |
| SH3BGRL2 | hsa-miR-27b-3p |
| GLI3 | hsa-miR-296-3p |
| COL11A1 | hsa-miR-29a-3p |
| ISG20L2 | hsa-miR-29a-3p |
| CDC7 | hsa-miR-29a-3p |
| FOS | hsa-miR-29a-3p |
| KCTD5 | hsa-miR-29a-3p |
| IFI30 | hsa-miR-29a-3p |
| DNMT3A | hsa-miR-29a-3p |
| COL5A2 | hsa-miR-29a-3p |
| COL3A1 | hsa-miR-29a-3p |
| MYBL2 | hsa-miR-29a-3p |
| GNB4 | hsa-miR-29a-3p |
| HMGCS1 | hsa-miR-29a-3p |
| TBC1D7 | hsa-miR-29a-3p |
| CALU | hsa-miR-29a-3p |
| LOXL2 | hsa-miR-29a-3p |
| COL11A1 | hsa-miR-29b-3p |
| ISG20L2 | hsa-miR-29b-3p |
| CDC7 | hsa-miR-29b-3p |
| FOS | hsa-miR-29b-3p |
| KCTD5 | hsa-miR-29b-3p |
| IFI30 | hsa-miR-29b-3p |
| DNMT3A | hsa-miR-29b-3p |
| COL5A2 | hsa-miR-29b-3p |
| COL3A1 | hsa-miR-29b-3p |
| MYBL2 | hsa-miR-29b-3p |
| GNB4 | hsa-miR-29b-3p |
| HMGCS1 | hsa-miR-29b-3p |
| TBC1D7 | hsa-miR-29b-3p |
| CALU | hsa-miR-29b-3p |
| LOXL2 | hsa-miR-29b-3p |
| COL11A1 | hsa-miR-29c-3p |
| ISG20L2 | hsa-miR-29c-3p |
| CDC7 | hsa-miR-29c-3p |
| FOS | hsa-miR-29c-3p |
| KCTD5 | hsa-miR-29c-3p |
| IFI30 | hsa-miR-29c-3p |
| DNMT3A | hsa-miR-29c-3p |
| COL5A2 | hsa-miR-29c-3p |
| COL3A1 | hsa-miR-29c-3p |
| MYBL2 | hsa-miR-29c-3p |
| GNB4 | hsa-miR-29c-3p |
| HMGCS1 | hsa-miR-29c-3p |
| TBC1D7 | hsa-miR-29c-3p |
| CALU | hsa-miR-29c-3p |
| LOXL2 | hsa-miR-29c-3p |
| F3 | hsa-miR-300 |
| GFRA1 | hsa-miR-300 |
| COL3A1 | hsa-miR-300 |
| EBF1 | hsa-miR-300 |
| HMBOX1 | hsa-miR-300 |
| CSTF2 | hsa-miR-300 |
| LRP8 | hsa-miR-301a-3p |
| DEPDC1 | hsa-miR-301a-3p |
| F3 | hsa-miR-301a-3p |
| S1PR1 | hsa-miR-301a-3p |
| CEP55 | hsa-miR-301a-3p |
| IGF1 | hsa-miR-301a-3p |
| NPTX1 | hsa-miR-301a-3p |
| INHBB | hsa-miR-301a-3p |
| LRIG1 | hsa-miR-301a-3p |
| KIT | hsa-miR-301a-3p |
| LRP8 | hsa-miR-301b-3p |
| DEPDC1 | hsa-miR-301b-3p |
| F3 | hsa-miR-301b-3p |
| S1PR1 | hsa-miR-301b-3p |
| CEP55 | hsa-miR-301b-3p |
| IGF1 | hsa-miR-301b-3p |
| NPTX1 | hsa-miR-301b-3p |
| INHBB | hsa-miR-301b-3p |
| LRIG1 | hsa-miR-301b-3p |
| KIT | hsa-miR-301b-3p |
| CAMK2N1 | hsa-miR-302a-3p |
| TMEM123 | hsa-miR-302a-3p |
| TMEM25 | hsa-miR-302a-3p |
| CA12 | hsa-miR-302a-3p |
| KPNA2 | hsa-miR-302a-3p |
| SDC1 | hsa-miR-302a-3p |
| RND3 | hsa-miR-302a-3p |
| SLC40A1 | hsa-miR-302a-3p |
| TIPARP | hsa-miR-302a-3p |
| CAMK2N1 | hsa-miR-302b-3p |
| TMEM123 | hsa-miR-302b-3p |
| TMEM25 | hsa-miR-302b-3p |
| CA12 | hsa-miR-302b-3p |
| KPNA2 | hsa-miR-302b-3p |
| SDC1 | hsa-miR-302b-3p |
| RND3 | hsa-miR-302b-3p |
| SLC40A1 | hsa-miR-302b-3p |
| TIPARP | hsa-miR-302b-3p |
| CAMK2N1 | hsa-miR-302c-3p |
| TMEM123 | hsa-miR-302c-3p |
| TMEM25 | hsa-miR-302c-3p |
| KPNA2 | hsa-miR-302c-3p |
| SDC1 | hsa-miR-302c-3p |
| RND3 | hsa-miR-302c-3p |
| SLC40A1 | hsa-miR-302c-3p |
| TIPARP | hsa-miR-302c-3p |
| CAMK2N1 | hsa-miR-302d-3p |
| TMEM123 | hsa-miR-302d-3p |
| TMEM25 | hsa-miR-302d-3p |
| CA12 | hsa-miR-302d-3p |
| KPNA2 | hsa-miR-302d-3p |
| SDC1 | hsa-miR-302d-3p |
| RND3 | hsa-miR-302d-3p |
| SLC40A1 | hsa-miR-302d-3p |
| TIPARP | hsa-miR-302d-3p |
| CAMK2N1 | hsa-miR-302e |
| TMEM123 | hsa-miR-302e |
| TMEM25 | hsa-miR-302e |
| CA12 | hsa-miR-302e |
| KPNA2 | hsa-miR-302e |
| SDC1 | hsa-miR-302e |
| RND3 | hsa-miR-302e |
| SLC40A1 | hsa-miR-302e |
| TIPARP | hsa-miR-302e |
| CAMK2N1 | hsa-miR-30a-5p |
| CDC7 | hsa-miR-30a-5p |
| FLVCR1 | hsa-miR-30a-5p |
| DDIT4 | hsa-miR-30a-5p |
| CAT | hsa-miR-30a-5p |
| KCTD5 | hsa-miR-30a-5p |
| FRZB | hsa-miR-30a-5p |
| MYBL2 | hsa-miR-30a-5p |
| AMOTL2 | hsa-miR-30a-5p |
| PTPN13 | hsa-miR-30a-5p |
| SOBP | hsa-miR-30a-5p |
| ARL4A | hsa-miR-30a-5p |
| RUNDC3B | hsa-miR-30a-5p |
| CALU | hsa-miR-30a-5p |
| LYN | hsa-miR-30a-5p |
| CTHRC1 | hsa-miR-30a-5p |
| CAMK2N1 | hsa-miR-30b-5p |
| CDC7 | hsa-miR-30b-5p |
| FLVCR1 | hsa-miR-30b-5p |
| DDIT4 | hsa-miR-30b-5p |
| CAT | hsa-miR-30b-5p |
| KCTD5 | hsa-miR-30b-5p |
| FRZB | hsa-miR-30b-5p |
| MYBL2 | hsa-miR-30b-5p |
| AMOTL2 | hsa-miR-30b-5p |
| PTPN13 | hsa-miR-30b-5p |
| SOBP | hsa-miR-30b-5p |
| ARL4A | hsa-miR-30b-5p |
| RUNDC3B | hsa-miR-30b-5p |
| CALU | hsa-miR-30b-5p |
| LYN | hsa-miR-30b-5p |
| CTHRC1 | hsa-miR-30b-5p |
| CAMK2N1 | hsa-miR-30c-5p |
| CDC7 | hsa-miR-30c-5p |
| FLVCR1 | hsa-miR-30c-5p |
| DDIT4 | hsa-miR-30c-5p |
| CAT | hsa-miR-30c-5p |
| KCTD5 | hsa-miR-30c-5p |
| FRZB | hsa-miR-30c-5p |
| MYBL2 | hsa-miR-30c-5p |
| AMOTL2 | hsa-miR-30c-5p |
| PTPN13 | hsa-miR-30c-5p |
| SOBP | hsa-miR-30c-5p |
| ARL4A | hsa-miR-30c-5p |
| RUNDC3B | hsa-miR-30c-5p |
| CALU | hsa-miR-30c-5p |
| LYN | hsa-miR-30c-5p |
| CTHRC1 | hsa-miR-30c-5p |
| CAMK2N1 | hsa-miR-30d-5p |
| CDC7 | hsa-miR-30d-5p |
| FLVCR1 | hsa-miR-30d-5p |
| DDIT4 | hsa-miR-30d-5p |
| CAT | hsa-miR-30d-5p |
| KCTD5 | hsa-miR-30d-5p |
| FRZB | hsa-miR-30d-5p |
| MYBL2 | hsa-miR-30d-5p |
| AMOTL2 | hsa-miR-30d-5p |
| PTPN13 | hsa-miR-30d-5p |
| SOBP | hsa-miR-30d-5p |
| ARL4A | hsa-miR-30d-5p |
| RUNDC3B | hsa-miR-30d-5p |
| CALU | hsa-miR-30d-5p |
| LYN | hsa-miR-30d-5p |
| CTHRC1 | hsa-miR-30d-5p |
| CAMK2N1 | hsa-miR-30e-5p |
| CDC7 | hsa-miR-30e-5p |
| FLVCR1 | hsa-miR-30e-5p |
| DDIT4 | hsa-miR-30e-5p |
| CAT | hsa-miR-30e-5p |
| KCTD5 | hsa-miR-30e-5p |
| FRZB | hsa-miR-30e-5p |
| MYBL2 | hsa-miR-30e-5p |
| AMOTL2 | hsa-miR-30e-5p |
| PTPN13 | hsa-miR-30e-5p |
| SOBP | hsa-miR-30e-5p |
| ARL4A | hsa-miR-30e-5p |
| RUNDC3B | hsa-miR-30e-5p |
| CALU | hsa-miR-30e-5p |
| LYN | hsa-miR-30e-5p |
| CTHRC1 | hsa-miR-30e-5p |
| KDELR2 | hsa-miR-31-5p |
| ANP32E | hsa-miR-32-5p |
| DDIT4 | hsa-miR-32-5p |
| DKK3 | hsa-miR-32-5p |
| DUSP6 | hsa-miR-32-5p |
| MTHFD2 | hsa-miR-32-5p |
| RRBP1 | hsa-miR-32-5p |
| AURKA | hsa-miR-32-5p |
| BAK1 | hsa-miR-32-5p |
| SOBP | hsa-miR-32-5p |
| MMP16 | hsa-miR-32-5p |
| KIF14 | hsa-miR-320a |
| S1PR1 | hsa-miR-320a |
| SDHC | hsa-miR-320a |
| MASTL | hsa-miR-320a |
| TMEM123 | hsa-miR-320a |
| FOXM1 | hsa-miR-320a |
| TIPARP | hsa-miR-320a |
| SOBP | hsa-miR-320a |
| GNAI1 | hsa-miR-320a |
| KIF14 | hsa-miR-320b |
| S1PR1 | hsa-miR-320b |
| SDHC | hsa-miR-320b |
| MASTL | hsa-miR-320b |
| TMEM123 | hsa-miR-320b |
| FOXM1 | hsa-miR-320b |
| TIPARP | hsa-miR-320b |
| SOBP | hsa-miR-320b |
| GNAI1 | hsa-miR-320b |
| KIF14 | hsa-miR-320c |
| S1PR1 | hsa-miR-320c |
| SDHC | hsa-miR-320c |
| MASTL | hsa-miR-320c |
| TMEM123 | hsa-miR-320c |
| FOXM1 | hsa-miR-320c |
| TIPARP | hsa-miR-320c |
| SOBP | hsa-miR-320c |
| GNAI1 | hsa-miR-320c |
| KIF14 | hsa-miR-320d |
| S1PR1 | hsa-miR-320d |
| SDHC | hsa-miR-320d |
| MASTL | hsa-miR-320d |
| TMEM123 | hsa-miR-320d |
| FOXM1 | hsa-miR-320d |
| TIPARP | hsa-miR-320d |
| SOBP | hsa-miR-320d |
| GNAI1 | hsa-miR-320d |
| INHBB | hsa-miR-329-3p |
| ARL4A | hsa-miR-329-3p |
| CALU | hsa-miR-335-5p |
| AMOTL2 | hsa-miR-338-3p |
| ARPC1B | hsa-miR-338-3p |
| DUSP6 | hsa-miR-33a-5p |
| MTHFD2 | hsa-miR-33a-5p |
| RNF128 | hsa-miR-33a-5p |
| DUSP6 | hsa-miR-33b-5p |
| MTHFD2 | hsa-miR-33b-5p |
| RNF128 | hsa-miR-33b-5p |
| F3 | hsa-miR-340-5p |
| ANP32E | hsa-miR-340-5p |
| S1PR1 | hsa-miR-340-5p |
| KCNK1 | hsa-miR-340-5p |
| ABAT | hsa-miR-340-5p |
| TOMM40 | hsa-miR-340-5p |
| LRIG1 | hsa-miR-340-5p |
| CCNG2 | hsa-miR-340-5p |
| ARL4A | hsa-miR-340-5p |
| DUSP6 | hsa-miR-342-3p |
| CA12 | hsa-miR-342-3p |
| TMEM25 | hsa-miR-34a-5p |
| INHBB | hsa-miR-34a-5p |
| TMEM25 | hsa-miR-34c-5p |
| INHBB | hsa-miR-34c-5p |
| INHBB | hsa-miR-362-3p |
| ARL4A | hsa-miR-362-3p |
| ANP32E | hsa-miR-363-3p |
| DDIT4 | hsa-miR-363-3p |
| DKK3 | hsa-miR-363-3p |
| DUSP6 | hsa-miR-363-3p |
| MTHFD2 | hsa-miR-363-3p |
| RRBP1 | hsa-miR-363-3p |
| AURKA | hsa-miR-363-3p |
| BAK1 | hsa-miR-363-3p |
| SOBP | hsa-miR-363-3p |
| ADAR | hsa-miR-365a-3p |
| ADD3 | hsa-miR-365a-3p |
| HMGCS1 | hsa-miR-365a-3p |
| ANP32E | hsa-miR-367-3p |
| DDIT4 | hsa-miR-367-3p |
| DKK3 | hsa-miR-367-3p |
| DUSP6 | hsa-miR-367-3p |
| MTHFD2 | hsa-miR-367-3p |
| RRBP1 | hsa-miR-367-3p |
| AURKA | hsa-miR-367-3p |
| BAK1 | hsa-miR-367-3p |
| SOBP | hsa-miR-367-3p |
| CAMK2N1 | hsa-miR-372-3p |
| TMEM123 | hsa-miR-372-3p |
| TMEM25 | hsa-miR-372-3p |
| CA12 | hsa-miR-372-3p |
| KPNA2 | hsa-miR-372-3p |
| SDC1 | hsa-miR-372-3p |
| RND3 | hsa-miR-372-3p |
| SLC40A1 | hsa-miR-372-3p |
| TIPARP | hsa-miR-372-3p |
| CAMK2N1 | hsa-miR-373-3p |
| TMEM123 | hsa-miR-373-3p |
| TMEM25 | hsa-miR-373-3p |
| CA12 | hsa-miR-373-3p |
| KPNA2 | hsa-miR-373-3p |
| SDC1 | hsa-miR-373-3p |
| RND3 | hsa-miR-373-3p |
| SLC40A1 | hsa-miR-373-3p |
| TIPARP | hsa-miR-373-3p |
| TMEM123 | hsa-miR-374a-5p |
| DUSP6 | hsa-miR-374a-5p |
| FOXO1 | hsa-miR-374a-5p |
| SPRY2 | hsa-miR-374a-5p |
| CA12 | hsa-miR-374a-5p |
| ANLN | hsa-miR-374a-5p |
| TMEM123 | hsa-miR-374b-5p |
| DUSP6 | hsa-miR-374b-5p |
| FOXO1 | hsa-miR-374b-5p |
| SPRY2 | hsa-miR-374b-5p |
| CA12 | hsa-miR-374b-5p |
| ANLN | hsa-miR-374b-5p |
| EZR | hsa-miR-376a-3p |
| EZR | hsa-miR-376b-3p |
| TGFBR3 | hsa-miR-376c-3p |
| KPNA2 | hsa-miR-376c-3p |
| LRP8 | hsa-miR-377-3p |
| KCTD12 | hsa-miR-377-3p |
| CA12 | hsa-miR-377-3p |
| CCNG2 | hsa-miR-377-3p |
| SOBP | hsa-miR-378a-3p |
| F3 | hsa-miR-381-3p |
| GFRA1 | hsa-miR-381-3p |
| COL3A1 | hsa-miR-381-3p |
| EBF1 | hsa-miR-381-3p |
| HMBOX1 | hsa-miR-381-3p |
| CSTF2 | hsa-miR-381-3p |
| AMOTL2 | hsa-miR-382-5p |
| FOS | hsa-miR-383-5p |
| CIRBP | hsa-miR-383-5p |
| ANP32E | hsa-miR-384 |
| HDGF | hsa-miR-384 |
| SH3BGRL2 | hsa-miR-384 |
| CAMK2N1 | hsa-miR-410-3p |
| KPNA2 | hsa-miR-411-5p |
| DUSP1 | hsa-miR-411-5p |
| ABAT | hsa-miR-421 |
| MAGED1 | hsa-miR-421 |
| SOBP | hsa-miR-422a |
| TGFBR3 | hsa-miR-424-5p |
| HDGF | hsa-miR-424-5p |
| C1orf21 | hsa-miR-424-5p |
| CEP55 | hsa-miR-424-5p |
| CHEK1 | hsa-miR-424-5p |
| FGF7 | hsa-miR-424-5p |
| TMEM100 | hsa-miR-424-5p |
| AXIN2 | hsa-miR-424-5p |
| LRIG1 | hsa-miR-424-5p |
| FAT4 | hsa-miR-424-5p |
| ADRB2 | hsa-miR-424-5p |
| SH3BGRL2 | hsa-miR-424-5p |
| SOBP | hsa-miR-424-5p |
| ANLN | hsa-miR-424-5p |
| CALU | hsa-miR-424-5p |
| CA8 | hsa-miR-424-5p |
| HMBOX1 | hsa-miR-424-5p |
| STMN1 | hsa-miR-425-5p |
| LOXL2 | hsa-miR-425-5p |
| DDIT4 | hsa-miR-429 |
| ADD3 | hsa-miR-429 |
| ABAT | hsa-miR-429 |
| NPTX1 | hsa-miR-429 |
| RND3 | hsa-miR-429 |
| LRIG1 | hsa-miR-429 |
| AMOTL2 | hsa-miR-429 |
| PTPN13 | hsa-miR-429 |
| DUSP1 | hsa-miR-429 |
| GLI3 | hsa-miR-429 |
| ANLN | hsa-miR-429 |
| CALU | hsa-miR-429 |
| MMP16 | hsa-miR-429 |
| NMT2 | hsa-miR-433-3p |
| DDIT4 | hsa-miR-448 |
| ABAT | hsa-miR-448 |
| ETV1 | hsa-miR-448 |
| GNAI1 | hsa-miR-448 |
| TMEM25 | hsa-miR-449a |
| INHBB | hsa-miR-449a |
| TMEM25 | hsa-miR-449b-5p |
| INHBB | hsa-miR-449b-5p |
| LRP8 | hsa-miR-454-3p |
| DEPDC1 | hsa-miR-454-3p |
| F3 | hsa-miR-454-3p |
| CEP55 | hsa-miR-454-3p |
| IGF1 | hsa-miR-454-3p |
| NPTX1 | hsa-miR-454-3p |
| INHBB | hsa-miR-454-3p |
| LRIG1 | hsa-miR-454-3p |
| KIT | hsa-miR-454-3p |
| S1PR1 | hsa-miR-455-5p |
| ADD3 | hsa-miR-455-5p |
| RRM2 | hsa-miR-485-5p |
| SLC40A1 | hsa-miR-488-3p |
| HMGCS1 | hsa-miR-494-3p |
| GLI3 | hsa-miR-494-3p |
| PGK1 | hsa-miR-494-3p |
| DDIT4 | hsa-miR-495-3p |
| IGF1 | hsa-miR-495-3p |
| KPNA2 | hsa-miR-495-3p |
| DNMT3A | hsa-miR-495-3p |
| HMGCS1 | hsa-miR-495-3p |
| DUSP1 | hsa-miR-495-3p |
| TGFBR3 | hsa-miR-497-5p |
| HDGF | hsa-miR-497-5p |
| C1orf21 | hsa-miR-497-5p |
| CEP55 | hsa-miR-497-5p |
| CHEK1 | hsa-miR-497-5p |
| FGF7 | hsa-miR-497-5p |
| TMEM100 | hsa-miR-497-5p |
| AXIN2 | hsa-miR-497-5p |
| LRIG1 | hsa-miR-497-5p |
| FAT4 | hsa-miR-497-5p |
| ADRB2 | hsa-miR-497-5p |
| SH3BGRL2 | hsa-miR-497-5p |
| SOBP | hsa-miR-497-5p |
| ANLN | hsa-miR-497-5p |
| CALU | hsa-miR-497-5p |
| CA8 | hsa-miR-497-5p |
| HMBOX1 | hsa-miR-497-5p |
| H2AFZ | hsa-miR-499a-5p |
| CHEK1 | hsa-miR-503-5p |
| FGF7 | hsa-miR-503-5p |
| ANLN | hsa-miR-503-5p |
| CHEK1 | hsa-miR-505-3p |
| ABAT | hsa-miR-505-3p |
| MAGED1 | hsa-miR-505-3p |
| ANP32E | hsa-miR-506-3p |
| DUSP6 | hsa-miR-506-3p |
| SPRY2 | hsa-miR-506-3p |
| RRBP1 | hsa-miR-506-3p |
| KDELR2 | hsa-miR-506-3p |
| GLI3 | hsa-miR-506-3p |
| CAMK2N1 | hsa-miR-519d-3p |
| F3 | hsa-miR-519d-3p |
| ADAR | hsa-miR-519d-3p |
| S1PR1 | hsa-miR-519d-3p |
| MASTL | hsa-miR-519d-3p |
| ATL3 | hsa-miR-519d-3p |
| TMEM123 | hsa-miR-519d-3p |
| TMEM25 | hsa-miR-519d-3p |
| KPNA2 | hsa-miR-519d-3p |
| RND3 | hsa-miR-519d-3p |
| SLC40A1 | hsa-miR-519d-3p |
| RRM2 | hsa-miR-519d-3p |
| LRIG1 | hsa-miR-519d-3p |
| TIPARP | hsa-miR-519d-3p |
| CCNG2 | hsa-miR-519d-3p |
| SOBP | hsa-miR-519d-3p |
| ETV1 | hsa-miR-519d-3p |
| ARL4A | hsa-miR-519d-3p |
| HMBOX1 | hsa-miR-519d-3p |
| RNF128 | hsa-miR-519d-3p |
| CAMK2N1 | hsa-miR-520a-3p |
| TMEM123 | hsa-miR-520a-3p |
| TMEM25 | hsa-miR-520a-3p |
| CA12 | hsa-miR-520a-3p |
| KPNA2 | hsa-miR-520a-3p |
| SDC1 | hsa-miR-520a-3p |
| RND3 | hsa-miR-520a-3p |
| SLC40A1 | hsa-miR-520a-3p |
| TIPARP | hsa-miR-520a-3p |
| CAMK2N1 | hsa-miR-520b |
| TMEM123 | hsa-miR-520b |
| TMEM25 | hsa-miR-520b |
| CA12 | hsa-miR-520b |
| KPNA2 | hsa-miR-520b |
| SDC1 | hsa-miR-520b |
| RND3 | hsa-miR-520b |
| SLC40A1 | hsa-miR-520b |
| TIPARP | hsa-miR-520b |
| CAMK2N1 | hsa-miR-520c-3p |
| TMEM123 | hsa-miR-520c-3p |
| TMEM25 | hsa-miR-520c-3p |
| CA12 | hsa-miR-520c-3p |
| KPNA2 | hsa-miR-520c-3p |
| SDC1 | hsa-miR-520c-3p |
| RND3 | hsa-miR-520c-3p |
| SLC40A1 | hsa-miR-520c-3p |
| TIPARP | hsa-miR-520c-3p |
| CAMK2N1 | hsa-miR-520d-3p |
| TMEM123 | hsa-miR-520d-3p |
| TMEM25 | hsa-miR-520d-3p |
| CA12 | hsa-miR-520d-3p |
| KPNA2 | hsa-miR-520d-3p |
| SDC1 | hsa-miR-520d-3p |
| RND3 | hsa-miR-520d-3p |
| SLC40A1 | hsa-miR-520d-3p |
| TIPARP | hsa-miR-520d-3p |
| CAMK2N1 | hsa-miR-520e |
| TMEM123 | hsa-miR-520e |
| TMEM25 | hsa-miR-520e |
| CA12 | hsa-miR-520e |
| KPNA2 | hsa-miR-520e |
| SDC1 | hsa-miR-520e |
| RND3 | hsa-miR-520e |
| SLC40A1 | hsa-miR-520e |
| TIPARP | hsa-miR-520e |
| UBE2E1 | hsa-miR-542-3p |
| NMT2 | hsa-miR-543 |
| ARL4A | hsa-miR-543 |
| SPRY2 | hsa-miR-590-5p |
| ANP32E | hsa-miR-613 |
| ADAR | hsa-miR-613 |
| RRBP1 | hsa-miR-613 |
| STC2 | hsa-miR-613 |
| KDELR2 | hsa-miR-613 |
| AP1S1 | hsa-miR-613 |
| PARP1 | hsa-miR-7-5p |
| DDIT4 | hsa-miR-7-5p |
| C20orf24 | hsa-miR-7-5p |
| GLI3 | hsa-miR-7-5p |
| CALU | hsa-miR-7-5p |
| CAMK2N1 | hsa-miR-873-5p |
| HDGF | hsa-miR-873-5p |
| ABAT | hsa-miR-873-5p |
| HMOX1 | hsa-miR-873-5p |
| PGK1 | hsa-miR-873-5p |
| HMGCS1 | hsa-miR-874-3p |
| STMN1 | hsa-miR-9-5p |
| DUSP6 | hsa-miR-9-5p |
| KCTD12 | hsa-miR-9-5p |
| MTHFD2 | hsa-miR-9-5p |
| ANP32E | hsa-miR-92a-3p |
| DDIT4 | hsa-miR-92a-3p |
| DKK3 | hsa-miR-92a-3p |
| DUSP6 | hsa-miR-92a-3p |
| MTHFD2 | hsa-miR-92a-3p |
| RRBP1 | hsa-miR-92a-3p |
| AURKA | hsa-miR-92a-3p |
| BAK1 | hsa-miR-92a-3p |
| SOBP | hsa-miR-92a-3p |
| MMP16 | hsa-miR-92a-3p |
| ANP32E | hsa-miR-92b-3p |
| DDIT4 | hsa-miR-92b-3p |
| DKK3 | hsa-miR-92b-3p |
| DUSP6 | hsa-miR-92b-3p |
| MTHFD2 | hsa-miR-92b-3p |
| RRBP1 | hsa-miR-92b-3p |
| AURKA | hsa-miR-92b-3p |
| BAK1 | hsa-miR-92b-3p |
| SOBP | hsa-miR-92b-3p |
| MMP16 | hsa-miR-92b-3p |
| CAMK2N1 | hsa-miR-93-5p |
| F3 | hsa-miR-93-5p |
| ADAR | hsa-miR-93-5p |
| S1PR1 | hsa-miR-93-5p |
| MASTL | hsa-miR-93-5p |
| ATL3 | hsa-miR-93-5p |
| TMEM123 | hsa-miR-93-5p |
| TMEM25 | hsa-miR-93-5p |
| KPNA2 | hsa-miR-93-5p |
| RND3 | hsa-miR-93-5p |
| SLC40A1 | hsa-miR-93-5p |
| RRM2 | hsa-miR-93-5p |
| LRIG1 | hsa-miR-93-5p |
| TIPARP | hsa-miR-93-5p |
| CCNG2 | hsa-miR-93-5p |
| SOBP | hsa-miR-93-5p |
| ETV1 | hsa-miR-93-5p |
| ARL4A | hsa-miR-93-5p |
| HMBOX1 | hsa-miR-93-5p |
| RNF128 | hsa-miR-93-5p |
| CAMK2N1 | hsa-miR-96-5p |
| DEPDC1 | hsa-miR-96-5p |
| RGS2 | hsa-miR-96-5p |
| DTL | hsa-miR-96-5p |
| FOXO1 | hsa-miR-96-5p |
| ABAT | hsa-miR-96-5p |
| RND3 | hsa-miR-96-5p |
| PDK1 | hsa-miR-96-5p |
| LRIG1 | hsa-miR-96-5p |
| AMOTL2 | hsa-miR-96-5p |
| EZR | hsa-miR-96-5p |
| TGFBR3 | hsa-miR-98-5p |
| COL5A2 | hsa-miR-98-5p |
| RRM2 | hsa-miR-98-5p |
| COL3A1 | hsa-miR-98-5p |
| TTLL4 | hsa-miR-98-5p |
| DUSP1 | hsa-miR-98-5p |
| ADRB2 | hsa-miR-98-5p |
| AP1S1 | hsa-miR-98-5p |
