# Supplementary material for: Development of a prognostic model based on the ceRNA network in Triple-Negative Breast cancer
Source: PeerJ. 2025 Feb 27;13:e19063. doi: 10.7717/peerj.19063 (PMC11874946; doi:10.7717/peerj.19063)
Supplement: Table S3 [file peerj-13-19063-s009.docx]

**TableS3 The relationships between CirRNA and miRNA**

| **CirRNAs** | **miRNAs** |
| --- | --- |
| hsa_circ_0000069 | hsa-miR-101-3p |
| hsa_circ_0000376 | hsa-miR-10a-5p |
| hsa_circ_0000376 | hsa-miR-10b-5p |
| hsa_circ_0000632 | hsa-miR-125a-5p |
| hsa_circ_0000632 | hsa-miR-125b-5p |
| hsa_circ_0000069 | hsa-miR-1269a |
| hsa_circ_0000069 | hsa-miR-1269b |
| hsa_circ_0000069 | hsa-miR-1270 |
| hsa_circ_0001666 | hsa-miR-1270 |
| hsa_circ_0000707 | hsa-miR-1270 |
| hsa_circ_0000069 | hsa-miR-1276 |
| hsa_circ_0001666 | hsa-miR-1276 |
| hsa_circ_0000376 | hsa-miR-1277-5p |
| hsa_circ_0001917 | hsa-miR-1287-5p |
| hsa_circ_0000515 | hsa-miR-1296-5p |
| hsa_circ_0000517 | hsa-miR-1296-5p |
| hsa_circ_0000518 | hsa-miR-1296-5p |
| hsa_circ_0000519 | hsa-miR-1296-5p |
| hsa_circ_0000520 | hsa-miR-1296-5p |
| hsa_circ_0005455 | hsa-miR-1301-3p |
| hsa_circ_0000515 | hsa-miR-1306-5p |
| hsa_circ_0000517 | hsa-miR-1306-5p |
| hsa_circ_0000518 | hsa-miR-1306-5p |
| hsa_circ_0005455 | hsa-miR-130a-3p |
| hsa_circ_0005455 | hsa-miR-130b-3p |
| hsa_circ_0001535 | hsa-miR-132-3p |
| hsa_circ_0001666 | hsa-miR-133a-3p |
| hsa_circ_0001666 | hsa-miR-133b |
| hsa_circ_0000069 | hsa-miR-135a-5p |
| hsa_circ_0005455 | hsa-miR-135a-5p |
| hsa_circ_0000069 | hsa-miR-135b-5p |
| hsa_circ_0005455 | hsa-miR-135b-5p |
| hsa_circ_0000376 | hsa-miR-136-5p |
| hsa_circ_0000707 | hsa-miR-136-5p |
| hsa_circ_0005455 | hsa-miR-137 |
| hsa_circ_0000069 | hsa-miR-140-5p |
| hsa_circ_0005455 | hsa-miR-140-5p |
| hsa_circ_0005455 | hsa-miR-149-5p |
| hsa_circ_0001535 | hsa-miR-153-3p |
| hsa_circ_0005455 | hsa-miR-186-5p |
| hsa_circ_0000511 | hsa-miR-1908-5p |
| hsa_circ_0000512 | hsa-miR-1908-5p |
| hsa_circ_0000514 | hsa-miR-1908-5p |
| hsa_circ_0000515 | hsa-miR-1908-5p |
| hsa_circ_0001806 | hsa-miR-193a-5p |
| hsa_circ_0000707 | hsa-miR-2114-3p |
| hsa_circ_0005455 | hsa-miR-2116-3p |
| hsa_circ_0001535 | hsa-miR-212-3p |
| hsa_circ_0000288 | hsa-miR-214-3p |
| hsa_circ_0001633 | hsa-miR-218-5p |
| hsa_circ_0005455 | hsa-miR-218-5p |
| hsa_circ_0005455 | hsa-miR-223-3p |
| hsa_circ_0005455 | hsa-miR-2278 |
| hsa_circ_0001666 | hsa-miR-2355-3p |
| hsa_circ_0005455 | hsa-miR-2355-5p |
| hsa_circ_0000069 | hsa-miR-2467-3p |
| hsa_circ_0000632 | hsa-miR-2681-3p |
| hsa_circ_0000707 | hsa-miR-2681-3p |
| hsa_circ_0005455 | hsa-miR-2681-5p |
| hsa_circ_0000376 | hsa-miR-27a-3p |
| hsa_circ_0000376 | hsa-miR-27b-3p |
| hsa_circ_0000288 | hsa-miR-296-3p |
| hsa_circ_0000511 | hsa-miR-296-5p |
| hsa_circ_0000512 | hsa-miR-296-5p |
| hsa_circ_0000514 | hsa-miR-296-5p |
| hsa_circ_0000515 | hsa-miR-296-5p |
| hsa_circ_0000376 | hsa-miR-299-5p |
| hsa_circ_0001666 | hsa-miR-300 |
| hsa_circ_0005455 | hsa-miR-301a-3p |
| hsa_circ_0005455 | hsa-miR-301b-3p |
| hsa_circ_0000632 | hsa-miR-302a-3p |
| hsa_circ_0000632 | hsa-miR-302b-3p |
| hsa_circ_0000632 | hsa-miR-302c-3p |
| hsa_circ_0000632 | hsa-miR-302d-3p |
| hsa_circ_0000632 | hsa-miR-302e |
| hsa_circ_0000091 | hsa-miR-30c-5p |
| hsa_circ_0001666 | hsa-miR-3127-5p |
| hsa_circ_0005455 | hsa-miR-3140-3p |
| hsa_circ_0000632 | hsa-miR-3173-5p |
| hsa_circ_0000069 | hsa-miR-320a |
| hsa_circ_0000376 | hsa-miR-320a |
| hsa_circ_0005455 | hsa-miR-320a |
| hsa_circ_0000069 | hsa-miR-320b |
| hsa_circ_0000376 | hsa-miR-320b |
| hsa_circ_0005455 | hsa-miR-320b |
| hsa_circ_0000069 | hsa-miR-320c |
| hsa_circ_0005455 | hsa-miR-320c |
| hsa_circ_0000069 | hsa-miR-320d |
| hsa_circ_0005455 | hsa-miR-320d |
| hsa_circ_0000511 | hsa-miR-326 |
| hsa_circ_0000512 | hsa-miR-326 |
| hsa_circ_0000514 | hsa-miR-326 |
| hsa_circ_0000515 | hsa-miR-326 |
| hsa_circ_0000517 | hsa-miR-326 |
| hsa_circ_0000518 | hsa-miR-326 |
| hsa_circ_0000515 | hsa-miR-328-3p |
| hsa_circ_0000517 | hsa-miR-328-3p |
| hsa_circ_0000518 | hsa-miR-328-3p |
| hsa_circ_0000519 | hsa-miR-328-3p |
| hsa_circ_0000520 | hsa-miR-328-3p |
| hsa_circ_0000376 | hsa-miR-330-3p |
| hsa_circ_0000511 | hsa-miR-330-5p |
| hsa_circ_0000512 | hsa-miR-330-5p |
| hsa_circ_0000514 | hsa-miR-330-5p |
| hsa_circ_0000515 | hsa-miR-330-5p |
| hsa_circ_0000517 | hsa-miR-330-5p |
| hsa_circ_0000518 | hsa-miR-330-5p |
| hsa_circ_0000376 | hsa-miR-338-3p |
| hsa_circ_0001917 | hsa-miR-338-3p |
| hsa_circ_0000376 | hsa-miR-339-5p |
| hsa_circ_0001633 | hsa-miR-345-3p |
| hsa_circ_0000069 | hsa-miR-345-5p |
| hsa_circ_0000376 | hsa-miR-345-5p |
| hsa_circ_0000069 | hsa-miR-34a-5p |
| hsa_circ_0000069 | hsa-miR-34c-5p |
| hsa_circ_0005455 | hsa-miR-3605-3p |
| hsa_circ_0005455 | hsa-miR-3605-5p |
| hsa_circ_0005455 | hsa-miR-361-5p |
| hsa_circ_0000511 | hsa-miR-3612 |
| hsa_circ_0000512 | hsa-miR-3612 |
| hsa_circ_0000514 | hsa-miR-3612 |
| hsa_circ_0000515 | hsa-miR-3612 |
| hsa_circ_0000288 | hsa-miR-3619-5p |
| hsa_circ_0005455 | hsa-miR-3666 |
| hsa_circ_0000632 | hsa-miR-3690 |
| hsa_circ_0005455 | hsa-miR-371a-3p |
| hsa_circ_0000632 | hsa-miR-372-3p |
| hsa_circ_0000632 | hsa-miR-373-3p |
| hsa_circ_0000376 | hsa-miR-374b-3p |
| hsa_circ_0000376 | hsa-miR-376c-3p |
| hsa_circ_0000069 | hsa-miR-378a-3p |
| hsa_circ_0000069 | hsa-miR-378b |
| hsa_circ_0000069 | hsa-miR-378c |
| hsa_circ_0000069 | hsa-miR-378d |
| hsa_circ_0000069 | hsa-miR-378e |
| hsa_circ_0000069 | hsa-miR-378f |
| hsa_circ_0000069 | hsa-miR-378h |
| hsa_circ_0000069 | hsa-miR-378i |
| hsa_circ_0001666 | hsa-miR-381-3p |
| hsa_circ_0000069 | hsa-miR-382-3p |
| hsa_circ_0005455 | hsa-miR-382-5p |
| hsa_circ_0001666 | hsa-miR-3918 |
| hsa_circ_0005455 | hsa-miR-3942-5p |
| hsa_circ_0000069 | hsa-miR-422a |
| hsa_circ_0005455 | hsa-miR-4295 |
| hsa_circ_0000632 | hsa-miR-4319 |
| hsa_circ_0000376 | hsa-miR-4428 |
| hsa_circ_0000069 | hsa-miR-4429 |
| hsa_circ_0005455 | hsa-miR-4429 |
| hsa_circ_0000069 | hsa-miR-449a |
| hsa_circ_0000069 | hsa-miR-449b-5p |
| hsa_circ_0000376 | hsa-miR-450b-5p |
| hsa_circ_0005455 | hsa-miR-454-3p |
| hsa_circ_0000069 | hsa-miR-4677-3p |
| hsa_circ_0005455 | hsa-miR-4703-5p |
| hsa_circ_0000069 | hsa-miR-4731-5p |
| hsa_circ_0000091 | hsa-miR-4766-3p |
| hsa_circ_0000288 | hsa-miR-485-5p |
| hsa_circ_0001666 | hsa-miR-486-5p |
| hsa_circ_0000376 | hsa-miR-489-3p |
| hsa_circ_0001666 | hsa-miR-493-5p |
| hsa_circ_0005455 | hsa-miR-500b-5p |
| hsa_circ_0005455 | hsa-miR-5047 |
| hsa_circ_0001666 | hsa-miR-506-5p |
| hsa_circ_0000091 | hsa-miR-5094 |
| hsa_circ_0000069 | hsa-miR-513c-5p |
| hsa_circ_0005455 | hsa-miR-513c-5p |
| hsa_circ_0005455 | hsa-miR-514a-3p |
| hsa_circ_0005455 | hsa-miR-514b-3p |
| hsa_circ_0000069 | hsa-miR-514b-5p |
| hsa_circ_0005455 | hsa-miR-514b-5p |
| hsa_circ_0005455 | hsa-miR-515-5p |
| hsa_circ_0000069 | hsa-miR-516b-5p |
| hsa_circ_0005455 | hsa-miR-519e-5p |
| hsa_circ_0000632 | hsa-miR-520a-3p |
| hsa_circ_0000632 | hsa-miR-520b |
| hsa_circ_0000632 | hsa-miR-520c-3p |
| hsa_circ_0000632 | hsa-miR-520d-3p |
| hsa_circ_0005455 | hsa-miR-520d-5p |
| hsa_circ_0000632 | hsa-miR-520e |
| hsa_circ_0001917 | hsa-miR-520f-3p |
| hsa_circ_0001806 | hsa-miR-520h |
| hsa_circ_0005455 | hsa-miR-524-5p |
| hsa_circ_0000376 | hsa-miR-526b-5p |
| hsa_circ_0005455 | hsa-miR-541-5p |
| hsa_circ_0000515 | hsa-miR-542-3p |
| hsa_circ_0000518 | hsa-miR-542-3p |
| hsa_circ_0000519 | hsa-miR-542-3p |
| hsa_circ_0000520 | hsa-miR-542-3p |
| hsa_circ_0005455 | hsa-miR-545-5p |
| hsa_circ_0000520 | hsa-miR-556-5p |
| hsa_circ_0001666 | hsa-miR-5579-3p |
| hsa_circ_0000707 | hsa-miR-5586-5p |
| hsa_circ_0001917 | hsa-miR-5586-5p |
| hsa_circ_0000069 | hsa-miR-561-5p |
| hsa_circ_0005455 | hsa-miR-561-5p |
| hsa_circ_0000288 | hsa-miR-5691 |
| hsa_circ_0005455 | hsa-miR-599 |
| hsa_circ_0000707 | hsa-miR-615-3p |
| hsa_circ_0000069 | hsa-miR-620 |
| hsa_circ_0001666 | hsa-miR-620 |
| hsa_circ_0000707 | hsa-miR-620 |
| hsa_circ_0005455 | hsa-miR-624-3p |
| hsa_circ_0000091 | hsa-miR-627-5p |
| hsa_circ_0000511 | hsa-miR-627-5p |
| hsa_circ_0000512 | hsa-miR-627-5p |
| hsa_circ_0000514 | hsa-miR-627-5p |
| hsa_circ_0000515 | hsa-miR-627-5p |
| hsa_circ_0000069 | hsa-miR-628-5p |
| hsa_circ_0000511 | hsa-miR-650 |
| hsa_circ_0000512 | hsa-miR-650 |
| hsa_circ_0000514 | hsa-miR-650 |
| hsa_circ_0000515 | hsa-miR-650 |
| hsa_circ_0000288 | hsa-miR-6509-3p |
| hsa_circ_0000707 | hsa-miR-6512-3p |
| hsa_circ_0000511 | hsa-miR-663a |
| hsa_circ_0000512 | hsa-miR-663a |
| hsa_circ_0000514 | hsa-miR-663a |
| hsa_circ_0000515 | hsa-miR-663a |
| hsa_circ_0000707 | hsa-miR-6720-5p |
| hsa_circ_0000511 | hsa-miR-6787-5p |
| hsa_circ_0000512 | hsa-miR-6787-5p |
| hsa_circ_0000514 | hsa-miR-6787-5p |
| hsa_circ_0000515 | hsa-miR-6787-5p |
| hsa_circ_0000632 | hsa-miR-6799-3p |
| hsa_circ_0000288 | hsa-miR-6805-3p |
| hsa_circ_0000707 | hsa-miR-6823-3p |
| hsa_circ_0005455 | hsa-miR-6835-3p |
| hsa_circ_0000288 | hsa-miR-6884-5p |
| hsa_circ_0005455 | hsa-miR-7-5p |
| hsa_circ_0000288 | hsa-miR-761 |
| hsa_circ_0000707 | hsa-miR-769-5p |
| hsa_circ_0005455 | hsa-miR-877-5p |
| hsa_circ_0000707 | hsa-miR-892c-5p |
| hsa_circ_0005455 | hsa-miR-892c-5p |
| hsa_circ_0000069 | hsa-miR-9-3p |
| hsa_circ_0001666 | hsa-miR-9-3p |
| hsa_circ_0005455 | hsa-miR-9-3p |
| hsa_circ_0000376 | hsa-miR-944 |
