# Supplementary material for: Development of a prognostic model based on the ceRNA network in Triple-Negative Breast cancer
Source: PeerJ. 2025 Feb 27;13:e19063. doi: 10.7717/peerj.19063 (PMC11874946; doi:10.7717/peerj.19063)
Supplement: Table S4 [file peerj-13-19063-s010.docx]

**Table S4 The expression profile of the CircRNAs**

| **CircRNA** | **logFC** | **Type** | **Regulation** | **chrome** | **Gene symbol** |
| --- | --- | --- | --- | --- | --- |
| hsa_circ_0005455 | 1.21 | Exonic | UP | Chr17 | KANSL1 |
| hsa_circ_0000632 | 1.08 | Exonic | UP | Chr15 | EDC3 |
| hsa_circ_0001666 | -3.54 | Exonic | Down | Chr6 | FAM120B |
| hsa_circ_0000069 | 1.03 | Exonic | UP | Chr1 | STIL |
