# Supplementary material for: Development of a prognostic model based on the ceRNA network in Triple-Negative Breast cancer
Source: PeerJ. 2025 Feb 27;13:e19063. doi: 10.7717/peerj.19063 (PMC11874946; doi:10.7717/peerj.19063)
Supplement: Table S5 [file peerj-13-19063-s011.docx]

**Table S5 The connection degree of each gene in the ceRNA network**

**based on GEO dataset**

| **Gene** | **Degree** | **Type** |
| --- | --- | --- |
| ABAT | 3 | mRNA |
| ADAR | 4 | mRNA |
| ADD3 | 4 | mRNA |
| AMOTL2 | 9 | mRNA |
| ANLN | 2 | mRNA |
| ANP32E | 5 | mRNA |
| AP1S1 | 4 | mRNA |
| ARHGAP11A | 2 | mRNA |
| ARL4A | 4 | mRNA |
| ARPC1B | 1 | mRNA |
| ATL3 | 2 | mRNA |
| AURKA | 1 | mRNA |
| AXIN2 | 2 | mRNA |
| BAIAP2L1 | 1 | mRNA |
| BAK1 | 3 | mRNA |
| C20orf24 | 2 | mRNA |
| CA12 | 11 | mRNA |
| CALU | 7 | mRNA |
| CAMK2N1 | 19 | mRNA |
| CAT | 1 | mRNA |
| CCNG2 | 5 | mRNA |
| CCT3 | 3 | mRNA |
| CDC7 | 1 | mRNA |
| CENPK | 1 | mRNA |
| CEP55 | 5 | mRNA |
| CNIH4 | 2 | mRNA |
| COL3A1 | 2 | mRNA |
| CSTF2 | 3 | mRNA |
| CTHRC1 | 1 | mRNA |
| CXCL12 | 2 | mRNA |
| DCDC2 | 1 | mRNA |
| DDIT4 | 8 | mRNA |
| DEPDC1 | 6 | mRNA |
| DKK3 | 1 | mRNA |
| DNMT3A | 4 | mRNA |
| DUSP1 | 4 | mRNA |
| DUSP6 | 1 | mRNA |
| EBF1 | 2 | mRNA |
| ELF3 | 1 | mRNA |
| ETV1 | 5 | mRNA |
| F3 | 12 | mRNA |
| FAT4 | 4 | mRNA |
| FLVCR1 | 5 | mRNA |
| FOS | 3 | mRNA |
| FOXM1 | 4 | mRNA |
| FOXO1 | 6 | mRNA |
| FRZB | 1 | mRNA |
| GFRA1 | 2 | mRNA |
| GLI3 | 3 | mRNA |
| GNAI1 | 5 | mRNA |
| H2AFV | 2 | mRNA |
| H2AFZ | 4 | mRNA |
| HDGF | 2 | mRNA |
| HMBOX1 | 4 | mRNA |
| HMGCS1 | 2 | mRNA |
| IGF1 | 6 | mRNA |
| INHBB | 11 | mRNA |
| IRX2 | 2 | mRNA |
| KCNK1 | 2 | mRNA |
| KCTD12 | 1 | mRNA |
| KCTD5 | 1 | mRNA |
| KDELR2 | 4 | mRNA |
| KIF14 | 4 | mRNA |
| KIT | 5 | mRNA |
| KLHL13 | 1 | mRNA |
| KPNA2 | 16 | mRNA |
| LOXL2 | 2 | mRNA |
| LRIG1 | 10 | mRNA |
| LRP8 | 5 | mRNA |
| LYN | 1 | mRNA |
| MASTL | 6 | mRNA |
| MMP16 | 1 | mRNA |
| MPZL1 | 1 | mRNA |
| MTHFD2 | 2 | mRNA |
| MYBL2 | 1 | mRNA |
| NAP1L5 | 2 | mRNA |
| NAV3 | 1 | mRNA |
| NPTX1 | 9 | mRNA |
| PARP1 | 1 | mRNA |
| PDGFA | 1 | mRNA |
| PDK1 | 2 | mRNA |
| PGK1 | 1 | mRNA |
| PTPN13 | 3 | mRNA |
| RAP2A | 2 | mRNA |
| RGS2 | 1 | mRNA |
| RND3 | 19 | mRNA |
| RNF128 | 2 | mRNA |
| RRBP1 | 3 | mRNA |
| RRM2 | 3 | mRNA |
| RUNDC3B | 1 | mRNA |
| S1PR1 | 11 | mRNA |
| SDC1 | 14 | mRNA |
| SDHC | 4 | mRNA |
| SH3BGRL2 | 2 | mRNA |
| SHMT2 | 1 | mRNA |
| SLC40A1 | 14 | mRNA |
| SOBP | 12 | mRNA |
| SPRY1 | 2 | mRNA |
| SPRY2 | 4 | mRNA |
| STC2 | 5 | mRNA |
| STMN1 | 4 | mRNA |
| TFPI | 2 | mRNA |
| TGFBR3 | 8 | mRNA |
| TIPARP | 19 | mRNA |
| TMEM123 | 24 | mRNA |
| TMEM25 | 20 | mRNA |
| UBE2E1 | 1 | mRNA |
| hsa_circ_0000069 | 14 | circRNA |
| hsa_circ_0000091 | 1 | circRNA |
| hsa_circ_0000288 | 2 | circRNA |
| hsa_circ_0000376 | 9 | circRNA |
| hsa_circ_0000515 | 1 | circRNA |
| hsa_circ_0000518 | 1 | circRNA |
| hsa_circ_0000519 | 1 | circRNA |
| hsa_circ_0000520 | 1 | circRNA |
| hsa_circ_0000632 | 14 | circRNA |
| hsa_circ_0000707 | 1 | circRNA |
| hsa_circ_0001535 | 3 | circRNA |
| hsa_circ_0001633 | 1 | circRNA |
| hsa_circ_0001666 | 4 | circRNA |
| hsa_circ_0001917 | 1 | circRNA |
| hsa_circ_0005455 | 19 | circRNA |
| ADAMTS9-AS1 | 6 | lncRNA |
| ADAMTS9-AS2 | 16 | lncRNA |
| ARHGAP5-AS1 | 4 | lncRNA |
| BRWD1-AS1 | 2 | lncRNA |
| CRNDE | 13 | lncRNA |
| DGUOK-AS1 | 2 | lncRNA |
| DIAPH2-AS1 | 6 | lncRNA |
| DIO3OS | 12 | lncRNA |
| EMX2OS | 3 | lncRNA |
| FBXL19-AS1 | 13 | lncRNA |
| FNDC1-IT1 | 3 | lncRNA |
| GRIK1-AS1 | 2 | lncRNA |
| KIF25-AS1 | 9 | lncRNA |
| LINC00028 | 2 | lncRNA |
| LINC00052 | 9 | lncRNA |
| LINC00092 | 2 | lncRNA |
| LINC00113 | 2 | lncRNA |
| LINC00261 | 17 | lncRNA |
| LINC00484 | 11 | lncRNA |
| MAGI2-AS3 | 12 | lncRNA |
| MAST4-AS1 | 2 | lncRNA |
| MCM3AP-AS1 | 21 | lncRNA |
| MEG3 | 24 | lncRNA |
| MIR22HG | 5 | lncRNA |
| MIR497HG | 4 | lncRNA |
| PVT1 | 10 | lncRNA |
| RRM1-AS1 | 2 | lncRNA |
| SNHG1 | 10 | lncRNA |
| SNHG12 | 12 | lncRNA |
| SNHG3 | 12 | lncRNA |
| THRB-IT1 | 10 | lncRNA |
| TPRG1-AS1 | 6 | lncRNA |
| VENTXP1 | 10 | lncRNA |
| WDFY3-AS2 | 17 | lncRNA |
| hsa-miR-101-3p | 12 | miRNA |
| hsa-miR-107 | 13 | miRNA |
| hsa-miR-10a-5p | 13 | miRNA |
| hsa-miR-10b-5p | 4 | miRNA |
| hsa-miR-125a-5p | 11 | miRNA |
| hsa-miR-125b-5p | 11 | miRNA |
| hsa-miR-129-5p | 21 | miRNA |
| hsa-miR-1297 | 21 | miRNA |
| hsa-miR-130a-3p | 11 | miRNA |
| hsa-miR-130b-3p | 11 | miRNA |
| hsa-miR-132-3p | 8 | miRNA |
| hsa-miR-133a-3p | 3 | miRNA |
| hsa-miR-133b | 3 | miRNA |
| hsa-miR-135a-5p | 17 | miRNA |
| hsa-miR-135b-5p | 7 | miRNA |
| hsa-miR-136-5p | 3 | miRNA |
| hsa-miR-137 | 10 | miRNA |
| hsa-miR-139-5p | 14 | miRNA |
| hsa-miR-140-5p | 16 | miRNA |
| hsa-miR-142-3p | 12 | miRNA |
| hsa-miR-146b-5p | 10 | miRNA |
| hsa-miR-149-5p | 6 | miRNA |
| hsa-miR-153-3p | 6 | miRNA |
| hsa-miR-17-5p | 26 | miRNA |
| hsa-miR-186-5p | 5 | miRNA |
| hsa-miR-193a-3p | 7 | miRNA |
| hsa-miR-206 | 19 | miRNA |
| hsa-miR-20b-5p | 26 | miRNA |
| hsa-miR-212-3p | 16 | miRNA |
| hsa-miR-217 | 15 | miRNA |
| hsa-miR-218-5p | 7 | miRNA |
| hsa-miR-22-3p | 14 | miRNA |
| hsa-miR-223-3p | 5 | miRNA |
| hsa-miR-23b-3p | 25 | miRNA |
| hsa-miR-24-3p | 21 | miRNA |
| hsa-miR-27a-3p | 27 | miRNA |
| hsa-miR-27b-3p | 14 | miRNA |
| hsa-miR-296-3p | 2 | miRNA |
| hsa-miR-300 | 7 | miRNA |
| hsa-miR-301a-3p | 11 | miRNA |
| hsa-miR-301b-3p | 16 | miRNA |
| hsa-miR-302a-3p | 10 | miRNA |
| hsa-miR-302b-3p | 10 | miRNA |
| hsa-miR-302c-3p | 9 | miRNA |
| hsa-miR-302d-3p | 10 | miRNA |
| hsa-miR-302e | 10 | miRNA |
| hsa-miR-30c-5p | 17 | miRNA |
| hsa-miR-320a | 12 | miRNA |
| hsa-miR-320b | 12 | miRNA |
| hsa-miR-320c | 11 | miRNA |
| hsa-miR-320d | 11 | miRNA |
| hsa-miR-338-3p | 18 | miRNA |
| hsa-miR-34a-5p | 3 | miRNA |
| hsa-miR-34c-5p | 3 | miRNA |
| hsa-miR-363-3p | 19 | miRNA |
| hsa-miR-372-3p | 10 | miRNA |
| hsa-miR-373-3p | 10 | miRNA |
| hsa-miR-376c-3p | 3 | miRNA |
| hsa-miR-378a-3p | 2 | miRNA |
| hsa-miR-381-3p | 7 | miRNA |
| hsa-miR-382-5p | 2 | miRNA |
| hsa-miR-422a | 2 | miRNA |
| hsa-miR-425-5p | 9 | miRNA |
| hsa-miR-429 | 21 | miRNA |
| hsa-miR-449a | 3 | miRNA |
| hsa-miR-449b-5p | 3 | miRNA |
| hsa-miR-454-3p | 10 | miRNA |
| hsa-miR-455-5p | 15 | miRNA |
| hsa-miR-485-5p | 2 | miRNA |
| hsa-miR-520a-3p | 10 | miRNA |
| hsa-miR-520b | 10 | miRNA |
| hsa-miR-520c-3p | 10 | miRNA |
| hsa-miR-520d-3p | 10 | miRNA |
| hsa-miR-520e | 10 | miRNA |
| hsa-miR-542-3p | 5 | miRNA |
| hsa-miR-590-5p | 8 | miRNA |
| hsa-miR-613 | 19 | miRNA |
| hsa-miR-7-5p | 6 | miRNA |
