# Supplementary material for: Development of a prognostic model based on the ceRNA network in Triple-Negative Breast cancer
Source: PeerJ. 2025 Feb 27;13:e19063. doi: 10.7717/peerj.19063 (PMC11874946; doi:10.7717/peerj.19063)
Supplement: Table S6 [file peerj-13-19063-s012.docx]

**Table S6 The connection degree of each gene in the ceRNA network**

**associated with MAPK pathway**

| **Gene** | **Degree** | **Type** |
| --- | --- | --- |
| CA12 | 11 | mRNA |
| GFRA1 | 2 | mRNA |
| NPTX1 | 9 | mRNA |
| hsa_circ_0000069 | 2 | circRNA |
| hsa_circ_0000632 | 11 | circRNA |
| hsa_circ_0001666 | 2 | circRNA |
| hsa_circ_0005455 | 7 | circRNA |
| ADAMTS9-AS1 | 1 | lncRNA |
| ADAMTS9-AS2 | 2 | lncRNA |
| CRNDE | 1 | lncRNA |
| DGUOK-AS1 | 1 | lncRNA |
| DIAPH2-AS1 | 1 | lncRNA |
| DIO3OS | 1 | lncRNA |
| FBXL19-AS1 | 1 | lncRNA |
| LINC00113 | 1 | lncRNA |
| LINC00261 | 4 | lncRNA |
| LINC00484 | 1 | lncRNA |
| MAGI2-AS3 | 1 | lncRNA |
| MCM3AP-AS1 | 3 | lncRNA |
| MEG3 | 3 | lncRNA |
| PVT1 | 1 | lncRNA |
| SNHG12 | 1 | lncRNA |
| SNHG3 | 2 | lncRNA |
| THRB-IT1 | 2 | lncRNA |
| TPRG1-AS1 | 1 | lncRNA |
| VENTXP1 | 1 | lncRNA |
| WDFY3-AS2 | 3 | lncRNA |
| hsa-miR-130a-3p | 2 | miRNA |
| hsa-miR-130b-3p | 2 | miRNA |
| hsa-miR-135a-5p | 13 | miRNA |
| hsa-miR-135b-5p | 3 | miRNA |
| hsa-miR-139-5p | 10 | miRNA |
| hsa-miR-300 | 2 | miRNA |
| hsa-miR-301a-3p | 2 | miRNA |
| hsa-miR-301b-3p | 7 | miRNA |
| hsa-miR-302a-3p | 2 | miRNA |
| hsa-miR-302b-3p | 2 | miRNA |
| hsa-miR-302d-3p | 2 | miRNA |
| hsa-miR-302e | 2 | miRNA |
| hsa-miR-372-3p | 2 | miRNA |
| hsa-miR-373-3p | 2 | miRNA |
| hsa-miR-381-3p | 2 | miRNA |
| hsa-miR-429 | 9 | miRNA |
| hsa-miR-454-3p | 2 | miRNA |
| hsa-miR-520a-3p | 2 | miRNA |
| hsa-miR-520b | 2 | miRNA |
| hsa-miR-520c-3p | 2 | miRNA |
| hsa-miR-520d-3p | 2 | miRNA |
| hsa-miR-520e | 2 | miRNA |
